# Adhesive Tape‐Inspired Polymer Coatings Enable Record‐Stable Zinc Anodes for High‐Rate Aqueous Batteries

**DOI:** 10.1002/advs.202520648

**Published:** 2025-12-12

**Authors:** Shuo Zhao, Shuyue Luo, Yangming Zhang, Rongyan Xie, Zhen Liu, Zhengyin Yao, Xianru He, Jiangbo Li, Xiang Yao, Zhou Zhou, Dongbai Sun, Peng Zhang

**Affiliations:** ^1^ Southern Marine Science and Engineering Guangdong Laboratory (Zhuhai), School of Materials Science and Engineering Sun Yat‐sen University Guangzhou 510275 China; ^2^ Medical Devices Research & Testing Center South China University of Technology Guangzhou 510006 China; ^3^ School of New Energy and Materials Southwest Petroleum University Chengdu 610500 China; ^4^ Engineering Center for Superlubricity Jihua Laboratory Foshan 528200 China; ^5^ JiangSu CheeShine Performance Materials Co., Ltd. Huaian 223200 China; ^6^ Center for Neutron Science and Technology, Guangdong Provincial Key Laboratory of Magnetoelectric Physics and Devices, School of Physics Sun Yat‐sen University Guangzhou 510275 China

**Keywords:** aqueous zinc batteries, dendrite suppression, ion‐selective transport, polymer coating, zinc anode protection

## Abstract

The development of stable zinc anodes is critical for advancing high‐rate, long‐life aqueous zinc batteries. Here a tape‐inspired anode modification is reported using a poly (methyl methacrylate)‐grafted natural rubber (NRAc) copolymer as a conformal protective coating. The microphase‐separated architecture integrates elastic, hydrophobic NR domains with Zn^2+^‐coordinating PMMA nanodomains, forming mechanically adaptive and ion‐selective channels. Density functional theory and microscopy reveal that Zn^2+^ preferentially binds to PMMA, lowering desolvation barriers and biasing deposition toward the stable (002) facet. This dual mechanical‐chemical regulation suppresses dendrite growth and parasitic hydrogen evolution, while maintaining intimate interfacial contact under dynamic cycling. As a result, NRAc@Zn symmetric cells achieve unprecedented cycling lifetimes exceeding 32,000 cycles, while Zn||V_2_O_5_ full cells deliver stable operation at current densities up to 10 A g^−1^. Scaling to a 1.5 Ah pouch cell demonstrates the practical feasibility of the approach. This work establishes polymer microphase engineering, inspired by the multifunctionality of adhesive tapes, as a versatile strategy to stabilize Zn anodes and advance the deployment of aqueous Zn batteries for large‐scale energy storage.

## Graphical abstract

An adhesive tape–inspired anode stabilization strategy is proposed by grafting poly(methyl methacrylate) onto natural rubber. The resulting copolymer coating exhibits elasticity, adhesion, and ion‐selective nanodomains, which lower Zn^2+^ desolvation barriers, guide deposition to the stable (002) facet, and ensure intimate interfacial contact, delivering over 32,000 stable cycles in symmetric Zn cells.

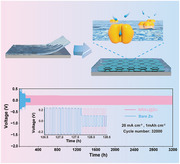

## 1 Introduction

Rechargeable aqueous zinc‐ion batteries (ZIBs) have gained significant attention as promising candidates for large‐scale energy storage due to the inherent advantages of metallic zinc anodes, such as their safety, low cost, high volumetric capacity, and environmental friendliness.^[^ ^1^, ^2^ ^]^ Recent advances in ZIB cathodes have substantially improved specific capacity, voltage windows, and cycling stability.^[^ ^3^, ^4^, ^5^ ^]^ However, the practical application of ZIBs, especially under high‐rate and high‐capacity conditions, remains hindered by persistent issues at the zinc metal anode. Notably, high current densities and long cycling periods exacerbate challenges such as dendritic growth, interface instability, and side reactions (e.g., hydrogen evolution, passivation), which significantly reduce battery life and performance.^[^ ^6^, ^7^, ^8^ ^]^ To address these limitations, recent works have focused on a variety of strategies, including electrolyte optimization, artificial solid‐electrolyte interphases, and anode surface modifications.^[^ ^9^, ^10^, ^11^ ^]^

Recent studies have demonstrated that nanoconfinement of Zn^2+^ transport within nanoscale polymeric or hybrid structures is a promising strategy to mitigate dendrite formation.^[^ ^12^, ^13^ ^]^ By confining ions within ordered nanodomains, one can effectively tune ion coordination and transport, facilitating accelerated desolvation and uniform nucleation, both of which suppress dendrite growth.^[^ ^14^, ^15^, ^16^ ^]^ These pioneering works have laid the foundation for using nanoconfinement in polymeric electrolytes and solid interphases to achieve stable and uniform Zn plating/stripping, thus providing a promising solution to the challenges of high‐rate cycling.

Nature offers additional design inspiration. Natural rubber (NR), a sustainable biopolymer, combines flexibility, toughness, amphiphilic properties, and intrinsic adaptability.^[^ ^17^ ^]^ Widely used in pressure‐sensitive adhesives and self‐healing tapes, NR ensures intimate, conformal contact with rough surfaces while maintaining mechanical resilience under dynamic stress.^[^ ^18^ ^]^ Chemically grafting NR with poly (methyl methacrylate) (PMMA) forms a block copolymer with microphase‐separated nanostructures, where rubbery segments provide stretchability and adhesion, while polar PMMA nanodomains act as nanoconfined ion‐conducting pathways.^[^ ^19^, ^20^ ^]^ This unique structure simultaneously promotes Zn^2+^ desolvation and regulates ion transport, addressing both the adhesion and ion transport challenges at the anode interface.^[^ ^21^, ^22^, ^23^ ^]^

We report a tape‐inspired anode modification strategy using a PMMA‐grafted natural rubber copolymer (NRAc), which combines the microphase‐separated structure of NR and PMMA to impart both mechanical flexibility and ion transport regulation. In this copolymer, the soft NR segments ensure stretchability and adhesion, while the PMMA nanodomains serve as zincophilic pathways that enhance Zn^2+^ transport and nucleation control.^[^ ^24^ ^]^ This synergy leads to ultrastable cycling in symmetric Zn cells, achieving over 3000 hours of stable operation at a high current density of 20 mA cm^−2^, and robust performance in pouch cells at practical capacities and high rates. The simplicity, scalability, and sustainability of this bio‐derived elastomer make it a powerful candidate for next‐generation high‐rate, long‐life Zn batteries.

## 2 Results and Discussion

### 2.1 Tape‐Inspired Multifunctional Design of NRAc

**Figure 1:**
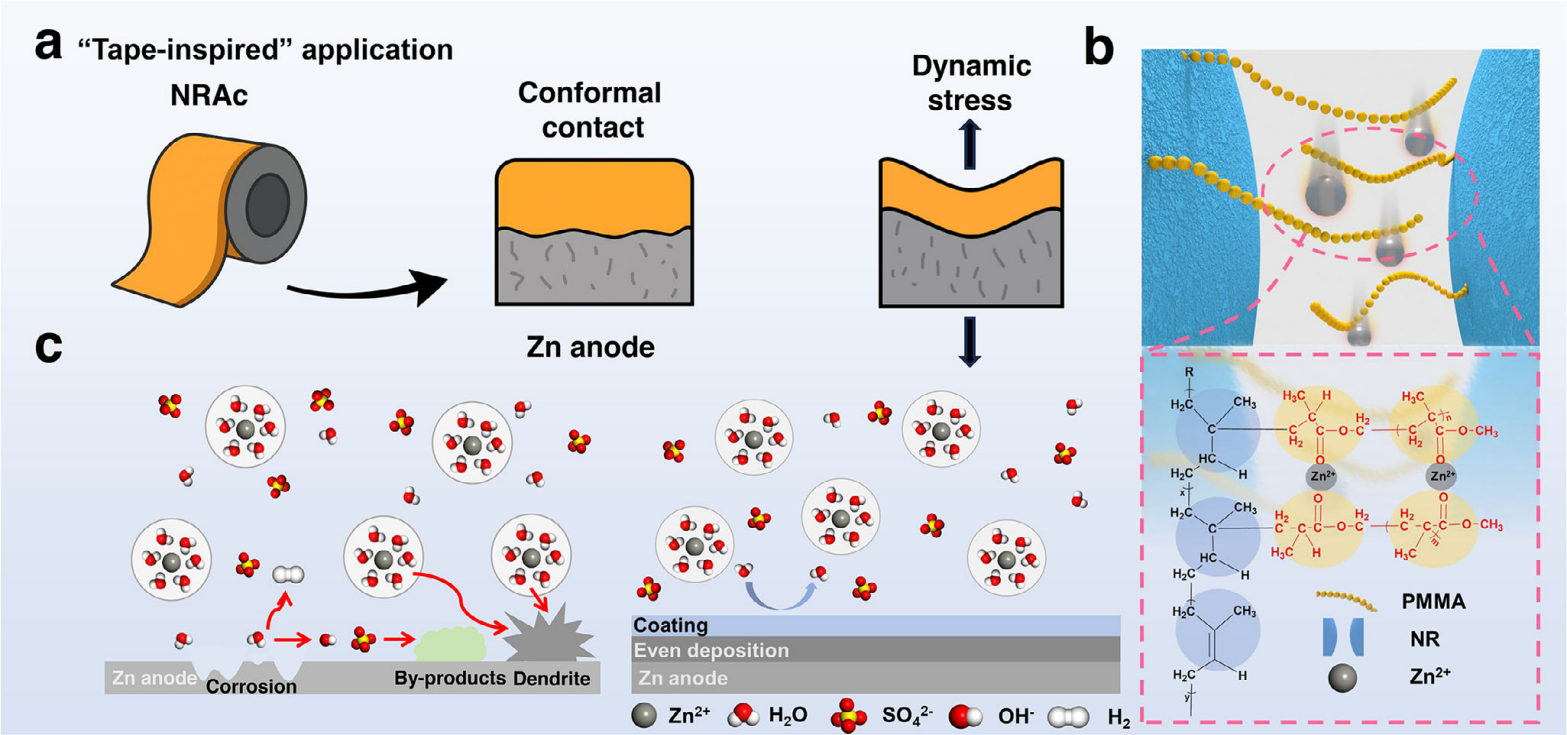
Tape‐inspired multifunctional NRAc coating on Zn anodes. a) Adhesive‐tape‐like mechanical properties, enabling conformal coverage and strong adhesion to Zn. b) Schematic of ion transport in NRAc, where microphase‐separated NR (hydrophobic) and PMMA (polar) microdomains form nanoconfined channels. Zn^2+^ preferentially coordinates with PMMA via carbonyl oxygens, ensuring uniform transport and deposition. c) Regulation mechanism of Zn plating/stripping by NRAc, showing suppression of side reactions and dendrite growth.

Inspired by the multifunctional adhesive properties of everyday tapes, we developed a PMMA‐grafted natural rubber (NRAc) coating as a conformal and multifunctional protective layer for Zn anodes (**Figure** **1**a). The natural rubber (NR) backbone provides mechanical elasticity and intrinsic hydrophobicity, enabling the coating to conform intimately to the rough Zn surface while resisting electrolyte penetration. This dual function ensures both physical compliance under repeated plating/stripping and corrosion resistance by limiting water‐induced side reactions at the metal surface.^[^ ^25^, ^26^ ^]^ Meanwhile, the PMMA side chains introduce polar carbonyl groups that selectively coordinate Zn^2+^, giving rise to preferential interactions at the electrolyte‐anode interface.^[^ ^25^ ^]^ Together, NR and PMMA form a microphase‐separated structure in which elastic NR‐rich domains act as a flexible scaffold, while PMMA‐rich domains organize into polar, Zn^2+^‐affinitive nanochannels (Figure 1b). These nanoconfined pathways facilitate directional ion transport and reduce desolvation barriers, thereby promoting uniform Zn deposition.^[^ ^27^ ^]^

Such dual mechanical‐chemical regulation allows NRAc to act as a tape‐like interfacial layer that homogenizes Zn^2+^ flux, suppresses dendrite nucleation, and mitigates parasitic hydrogen evolution and surface passivation (Figure 1c). This strategy builds on earlier work with PMMA‐based protective layers, such as the study by Park et al., who demonstrated that PMMA/Zn salt hybrid coatings could suppress dendrites and side reactions by forming dense ion‐conductive networks.^[^ ^28^ ^]^ In contrast, NRAc advances this concept by integrating PMMA nanodomains into an NR backbone, thereby combining ionic selectivity with mechanical resilience and interfacial stability‐key requirements for long‐term, high‐rate cycling.^[^ ^29^, ^30^ ^]^

The preparation process of NRAc@Zn electrodes is illustrated in Figure S1 (Supporting Information). The rotational speed of the spin coater affects the electrochemical performance to some extent. We optimized the optimal speed through EIS testing (Figure S2, Supporting Information). To highlight the coating morphology and interfacial contact, the sample (≈7 µm) was examined by cross‐sectional FESEM and elemental mapping, confirming seamless adhesion to Zn foil and homogeneous elemental distribution across the interface (Figure S3, Supporting Information). FTIR spectra further verify the coexistence of NR and PMMA components, with characteristic carbonyl stretching bands of PMMA and hydrocarbon vibrations of NR clearly identified (Figure S4, Supporting Information).

**Figure 2:**
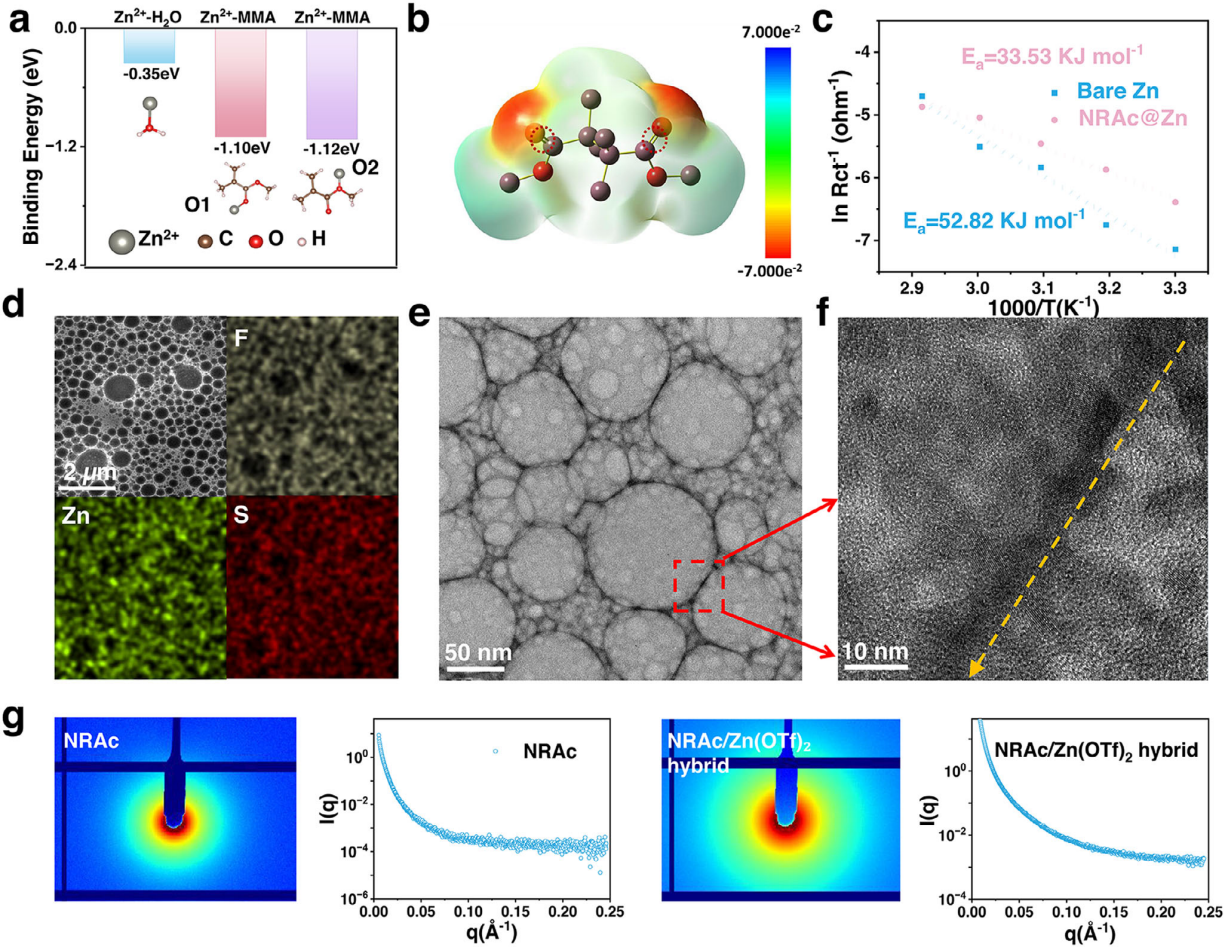
Zn^2+^coordination and transport dynamics regulated by NRAc. a) DFT binding energies of Zn^2+^with H_2_O and MMA oxygen atoms. b) Electrostatic potential distribution of MMA, highlighting carbonyl oxygens as binding sites. c) Arrhenius plots of symmetric Zn||Zn cells with and without NRAc, showing lower activation energy for Zn^2+^desolvation/transfer at the NRAc interface. d) HAADF‐STEM dark‐field image and elemental mapping of Zn(OTf)_2_/NRAc hybrid (Zn:MMA molar ratio 2:1). e) HAADF‐STEM bright‐field image showing microphase‐separated morphology: large NR‐rich domains (light) surrounded by narrow PMMA/Zn(OTf)_2_ ‐enriched edges (dark), consistent with the PMMA:NR weight ratio of 30:100. f) Enlarged bright‐field image of PMMA‐rich domains, showing crystalline Zn(OTf)_2_ lattice fringes forming continuous channel‐like structures (∼5 nm wide, arrowed) across the matrix (marked with arrow). g) SAXS profiles of pristine NRAc and Zn(OTf)_2_/NRAc hybrid, confirming preservation of microphase‐separated nanostructure.

Importantly, the microphase‐separated morphology of NRAc not only confirms the coexistence of elastic and polar domains but also foreshadows the formation of nanoconfined Zn^2+^‐transport channels, which will be further elucidated by atomic force microscopy (AFM), high‐angle annular dark‐field scanning transmission electron microscopy (HAADF‐STEM) and small‐angle X‐ray scattering (SAXS) analysis in the following section (**Figure** **2**). These findings establish NRAc as a rationally designed, tape‐inspired coating that integrates elasticity, adhesion, and ion coordination into a single multifunctional layer.

### 2.2 Zn^2+^ Coordination and Nanoconfined Ion Transport

To understand how NRAc regulates Zn^2+^ transport at the anode interface, we first evaluated the intrinsic affinity of PMMA segments toward Zn^2+^ using DFT calculations. The binding energy of Zn^2+^ to the carbonyl oxygen of MMA was markedly stronger than to water molecules (Figure 2a), underscoring the preferential coordination of Zn^2+^ with polar ester groups.^[^ ^31^ ^]^ The electrostatic potential distribution further highlighted carbonyl oxygens as the dominant coordination sites (Figure 2b). These results establish that PMMA domains act as Zn^2+^‐selective binding regions, consistent with a “like‐dissolves‐like” mechanism, where polar groups stabilize Zn^2+^ while suppressing competitive solvation by water.^[^ ^32^ ^]^ DFT calculations incorporating explicit solvation show that the [Zn(H_2_O)_5_]^2+^‐MMA binding energy (‐0.78 eV) is only slightly smaller than the gas‐phase Zn^2+^‐MMA value (‐1.10 eV), indicating that first‐shell hydration does not diminish the zincophilic interaction of MMA (Figure S5, Supporting Information). This supports a desolvation‐facilitated Zn^2+^ transport mechanism at the NRAc interface. These results support a desolvation‐facilitated mechanism at the NRAc interface. Comparable preferential binding of Zn^2+^ to PMMA over H_2_O has also been observed by Lu et al., reinforcing the universality of this principle.^[^ ^33^ ^]^

Electrochemical analysis confirmed the impact of such preferential interactions on interfacial ion dynamics. Symmetric‐cell EIS revealed a significantly reduced activation energy (*E*_a_) for Zn^2+^ transport across NRAc‐coated interfaces (33.5 kJ mol^−1^) compared with bare Zn (52.8 kJ mol^−1^, Figures 2c; S6, Supporting Information), reflecting facilitated desolvation and transfer within PMMA‐rich channels.^[^ ^34^ ^]^ Chronoamperometry at a fixed overpotential further showed stable and steady nucleation currents for NRAc@Zn, in contrast to the sharp current spikes characteristic of uncontrolled Zn nucleation on bare Zn (Figure S7, Supporting Information).^[^ ^35^ ^]^ In addition, comparative tests using NR‐only and PMMA‐only coatings (Figure S8, Supporting Information) show that neither component alone can deliver stable cycling. The NR coating exhibits high polarization due to limited Zn^2+^ transport, while the PMMA and bare Zn cells fail rapidly. These findings confirm that the synergistic NR‐PMMA microphase architecture in NRAc plays a vital role in enabling long‐term Zn anode stability.

Direct structural evidence of this synergy was provided by HAADF‐STEM and AFM imaging. In dark‐field mode (Figure 2d), clear microphase segregation was observed due to the thermodynamic incompatibility between NR and PMMA.^[^ ^36^ ^]^ The larger dark circular domains correspond to NR, consistent with its majority fraction in the NRAc copolymer (PMMA:NR weight ratio of 30:100). In contrast, PMMA/Zn(OTf)_2_ ‐enriched regions appeared brighter due to their higher electron density, and elemental mapping confirmed selective Zn accumulation within these polar domains (Zn:MMA molar ratio 2:1 in the hybrid). In bright‐field mode (Figure 2e), the NR‐rich domains appeared as light, rounded regions, surrounded by narrow, darker PMMA/Zn(OTf)_2_ edges. Upon magnification (Figure 2f), crystalline Zn(OTf)_2_ lattice fringes were observed within PMMA‐rich domains, forming continuous, channel‐like structures ≈5 nm in width that traversed the polymer matrix. These interconnected ionic pathways likely serve as directional conduits for Zn^2+^ migration, embedded within a mechanically robust NR framework that preserves elasticity.^[^ ^22^, ^29^, ^36^ ^]^ Similar microphase‐segregated features have also been confirmed using AFM in the neat NRAc sample (Figure S9, supporting information). The nanoscale ordering was further supported by SAXS measurements, which showed preserved microphase separation after Zn salt incorporation (Figure 2g). The absence of sharp scattering peaks indicates that the PMMA/Zn(OTf)_2_ domains adopt irregular, interconnected geometries rather than well‐ordered lamellae or cylinders‐consistent with the irregular morphologies observed in HAADF‐STEM images (Figure 2d–f). Similar irregularity of NR‐rich domains was also evident, reflecting the heterogeneous but functionally synergistic nature of the microstructure.^[^ ^29^, ^37^, ^38^ ^]^

Taken together, these results demonstrate that NRAc simultaneously lowers the desolvation barrier and constructs nanoconfined, continuous Zn^2+^ channels. This dual function reconciles fast ion transport with uniform nucleation, a capability that has previously required either sophisticated block copolymer architectures or rigid inorganic scaffolds.^[^ ^39^ ^]^ Here, such regulation is achieved within a scalable, rubber‐based interfacial coating, uniting mechanical resilience with ionic selectivity in a single tape‐inspired design.

### 2.3 Crystallographic Regulation of Zn Deposition

**Figure 3:**
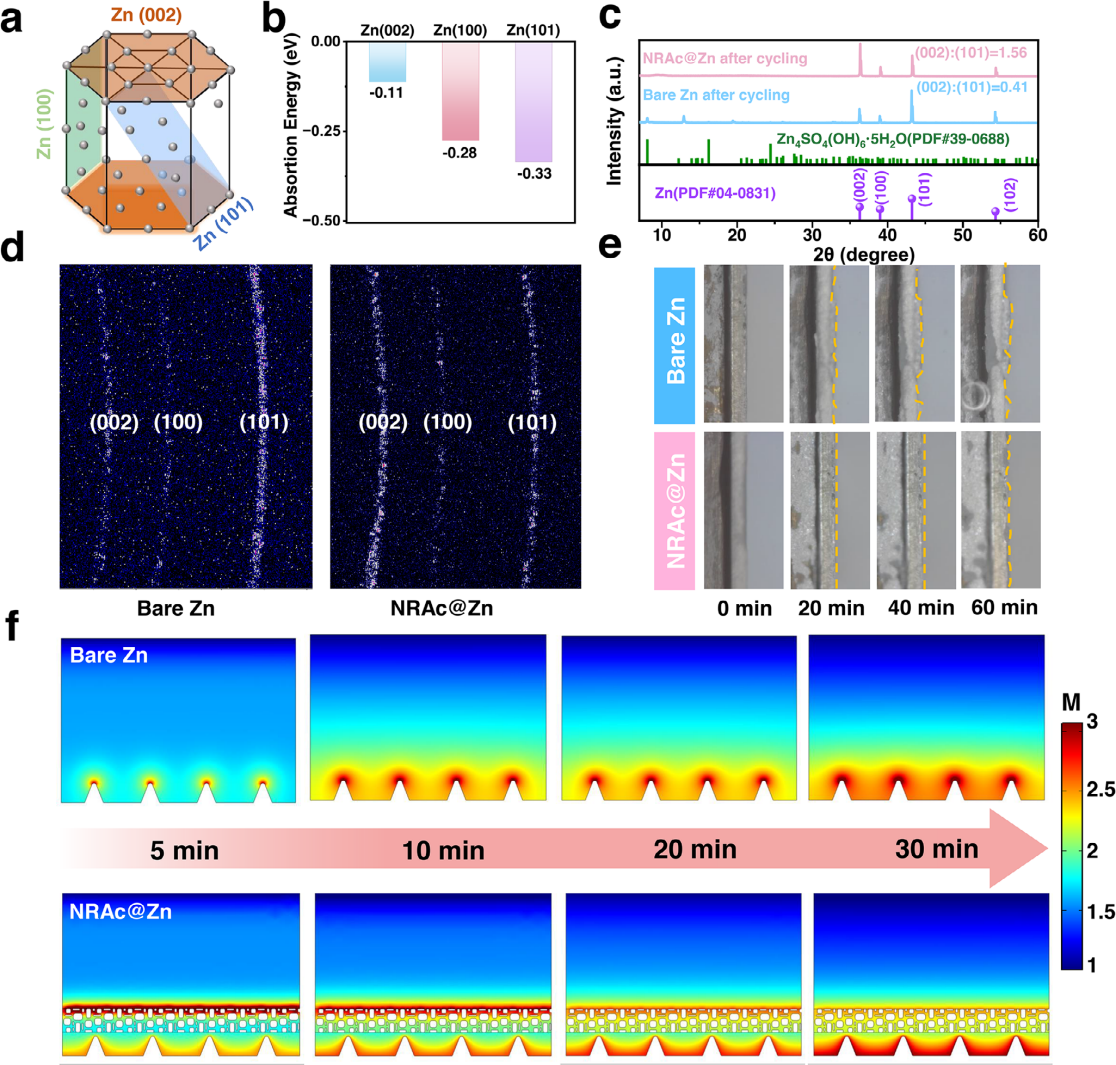
Crystallographic regulation of Zn deposition by NRAc. a) Hexagonal close‐packed Zn unit cell. b) Adsorption energies of MMA on Zn (002), Zn (100), and Zn (101) planes. c) XRD of Zn plated at 10 mA cm^−2^ for 1 h, with reference patterns. d) GIWAXS patterns highlighting (002) orientation for NRAc@Zn. e) In situ optical microscopy of Zn plating at 10 mA cm^−2^, showing dendrite‐mitigated growth on NRAc@Zn. Dashed lines were included to guide to eye. f) COMSOL simulations of electric field distributions, confirming homogenized Zn^2+^ transport under NRAc coating.

Beyond accelerating ion transport, the NRAc coating also exerts strong control over Zn crystallography during electrodeposition. DFT adsorption models showed that MMA molecules bind preferentially to Zn (100) and Zn (101) facets, while interacting only weakly with the close‐packed (002) facet (**Figures** **3**a and b; S10, Supporting Information). This selective interaction suppresses nucleation on high‐energy planes and biases growth toward the thermodynamically stable (002) orientation.^[^ ^40^ ^]^ Such facet control is particularly important, as Zn (002) is less prone to anisotropic stress accumulation and dendritic tip amplification, providing a crystallographic pathway for dendrite suppression.^[^ ^41^ ^]^

Structural characterization provides direct confirmation of theoretical insights. Ex situ XRD analysis reveals that the (002) reflection of the NRAc@Zn electrode intensifies progressively with deposition time, whereas the bare Zn electrode gradually loses (002) orientation and develops additional peaks associated with non‐preferred growth and corrosion (Figures 3c; S11, Supporting Information). GIWAXS patterns further confirmed the dominance of (002) orientation in NRAc@Zn, underscoring the coating's ability to bias Zn crystallization (Figure 3d).^[^ ^42^, ^43^ ^]^ Complementary in situ optical microscopy provided dynamic visualization of deposition behavior. For bare Zn, dashed guide lines were used to mark the plating front: the interface began to distort noticeably after only 20 min, with progressively severe warping through 40–60 min, coinciding with dendritic protrusions and localized bubble formation (Figure 3e). By contrast, the NRAc‐coated electrode maintained a flat, undistorted interface over the entire observation period, consistent with uniform deposition. These observations confirm that NRAc not only regulates ion flux but also directs crystallographic growth, jointly suppressing morphological instabilities.

The mechanistic basis was further validated by COMSOL simulations. Bare Zn exhibited highly localized electric field intensities and steep Zn^2+^ concentration gradients near protrusions, accelerating dendritic amplification via classical tip effects.^[^ ^33^ ^]^ In contrast, the NRAc coating homogenized both the electric field and Zn^2+^ flux, eliminating interfacial hotspots and promoting planar growth consistent with preferential (002) orientation (Figures 3f; S12, Supporting Information). These findings establish a clear structure‐property correlation: PMMA coordination sites create nanoconfined Zn^2+^ channels that not only lower desolvation barriers (Figure 2) but also bias nucleation toward the dendrite‐resistant (002) plane. This dual regulation ensures smooth, dendrite‐mitigated plating even under high current densities up to 60 mA cm^−2^, as mentioned in the following section.

### 2.4 Mechanical Resilience and Interfacial Stability

**Figure 4:**
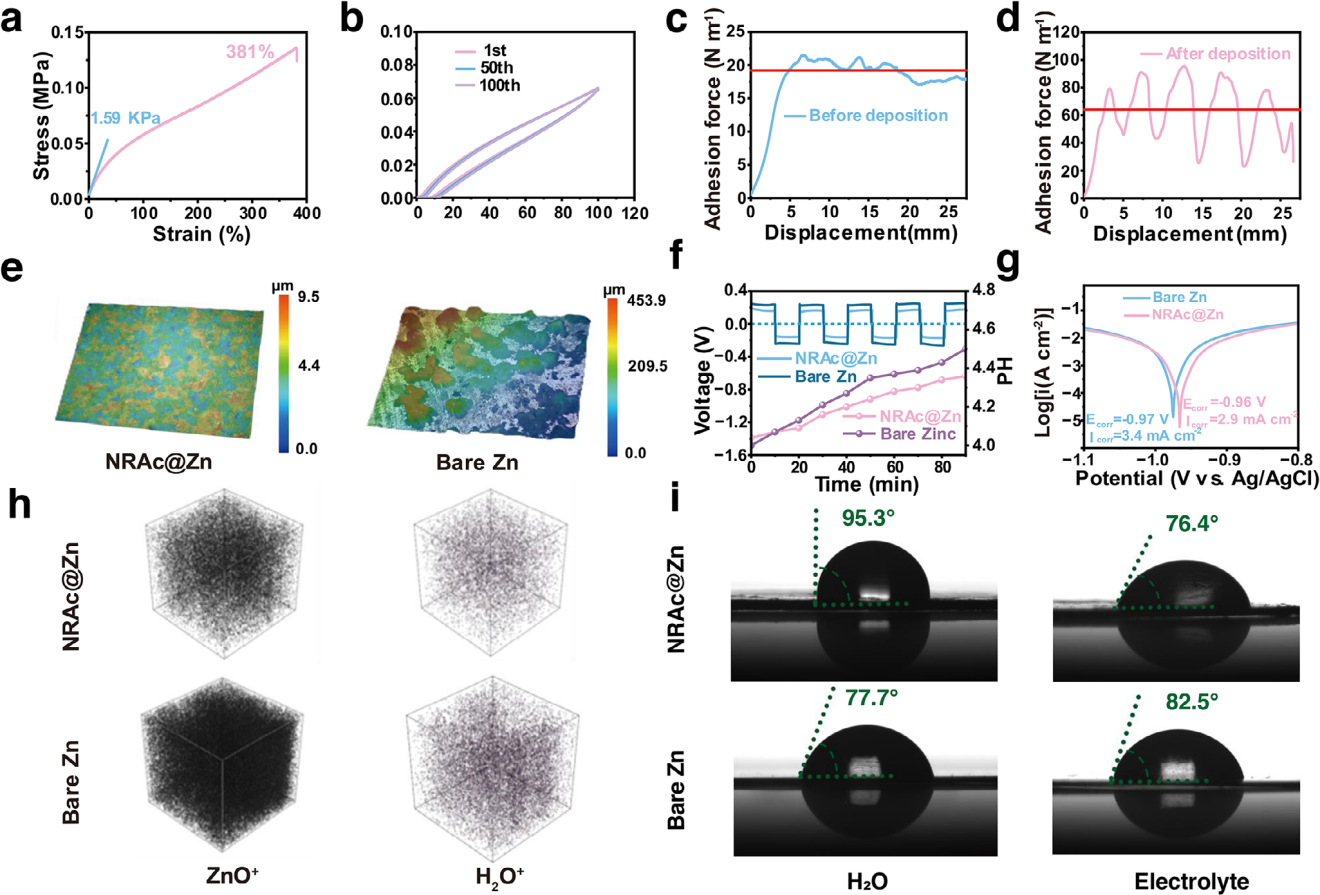
Mechanical, interfacial, and corrosion‐protective properties of NRAc. a) Stress‐strain curve of NRAc membrane, showing large elongation and low modulus. b) Stress‐strain curves after 1, 50, and 100 stretch‐release cycles, confirming mechanical resilience. c) Peel strength curves of NRAc‐coated Zn before and d) after deposition at 10 mA cm^−2^ (NRAc thickness: 0.5 mm). e) CLSM images of anodes in symmetric cells under alternating 0.2 V cycling (10 min intervals for 90 min). f) In situ pH monitoring of symmetric cells under alternating 0.2 V cycling (10 min intervals). g) Tafel plots comparing bare Zn and NRAc@Zn. h) TOF‐SIMS depth maps showing reduced ZnO^+^/H_2_O^+^ accumulation on NRAc@Zn after cycling. i) Contact angles of bare Zn and NRAc@Zn in water and 2 M Zn(OTf)_2_, showing balanced wetting/hydrophobicity.

A critical feature of the NRAC design lies in the elasticity imparted by the NR backbone, which enables the coating to accommodate dynamic surface fluctuations during Zn plating/stripping (Figure 1a). Tensile testing revealed a remarkable elongation of 381% and an ultra‐low Young's modulus of 1.59 kPa (**Figures** **4**a; S13, Supporting Information). Such softness allows the NRAc film to conform intimately to the zinc surface, effectively relieving local stress and suppressing crack initiation. Meanwhile, the strong interfacial adhesion derived from the synergistic interaction between the NR and PMMA components ensures that the coating remains firmly anchored to the substrate, maintaining continuous contact even under prolonged cycling. After 100 stretch–release cycles at 100% strain, the NRAc film retained its original resilience with only minor geometric distortion (Figure 4b), confirming its mechanical robustness and durable interfacial stability under repetitive deformation.

The adhesive nature of NRAc was further evidenced by peel‐strength measurements. On fresh Zn foil, the coating exhibited a strength of 19 N m^−1^ (Figure 4c), which increased markedly to 62 N m^−1^ after cycling in symmetric cells (Figure 4d). This enhancement highlights the self‐adaptability of the coating: as the Zn surface becomes roughened during cycling, the soft NRAc layer conforms more intimately to the evolving topography, increasing effective contact area and mechanical interlocking (Figure S14, Supporting Information). Such adaptive adhesion is reminiscent of commercial pressure‐sensitive tapes and recent ‘tape’‐like adhesives that combine low modulus, high stretchability, and strong interfacial contact for rough or wet surfaces.^[^ ^44^ ^]^ Importantly, confocal laser scanning microscopy (CLSM) revealed that NRAc@Zn surfaces remained smooth and intact after cycling (Figure 4e), in sharp contrast to the rough, cracked morphology of bare Zn. Together, these results demonstrate that NRAc not only forms conformal contact with pristine Zn but also dynamically accommodates interfacial changes, thereby stabilizing the anode surface against morphological degradation.

Electrochemical assessments corroborated these structural findings. In situ pH monitoring (Figure S16, Supporting Information) captured the interfacial environment under alternating 0.2 V cycling (10 min intervals). Both bare Zn and NRAc@Zn electrodes exhibited gradual increases in local pH (Figure 4f), but the rise was markedly smaller for NRAc@Zn. Since hydrogen evolution consumes protons and generates OH^−^, which induces precipitation of insulating byproducts such as Zn(OH)_2_ or zinc basic sulfates, the mitigated pH shift for NRAc@Zn reflects its suppression of parasitic reactions.^[^ ^45^ ^]^ Notably, the NRAc coating remains chemically stable against pH fluctuations during cycling, as evidenced by the unchanged FTIR signatures of the PMMA component (Figure S15, Supporting Information). Consistently, CLSM images of electrodes subjected to the pH‐monitoring protocol (Figure 4e) showed roughened and irregular bare Zn surfaces, while NRAc@Zn remained smooth and intact, highlighting its interfacial stabilization. To further quantify corrosion resistance, Tafel plots revealed lower corrosion currents for NRAc@Zn (2.96 mA cm^−2^) compared with bare Zn (3.40 mA cm^−2^), with nearly identical corrosion potentials (‐0.96 V vs. ‐0.97 V) (Figures 4g; S17, Supporting Information).^[^ ^46^ ^]^ Linear sweep voltammetry confirmed this trend, showing a higher hydrogen‐evolution overpotential for NRAc@Zn (Figure S18, Supporting Information), consistent with suppressed HER activity.

This protective effect was corroborated by TOF‐SIMS depth mapping, which revealed markedly reduced accumulation of ZnO^+^ and H_2_O^+^ species on NRAc@Zn compared with bare Zn (Figures 4h; S19, Supporting Information).^[^ ^47^ ^]^ Chemical stability was also evaluated in static electrolyte conditions. FESEM imaging showed that bare Zn developed cracks within hours of immersion in 2 M Zn(OTf)_2_, whereas NRAc‐protected Zn remained smooth (Figure S20, Supporting Information). Together, these results verify that NRAc acts as an effective interfacial barrier, simultaneously mitigating corrosion and suppressing side reactions.

To rationalize these behaviors, wettability was probed by contact‐angle measurements. NR domains were hydrophobic toward both water and Zn(OTf)_2_ electrolyte, while PMMA domains exhibited lower angles specifically with electrolyte (Figures 4i; S21, Supporting Information). This balanced hydrophobic‐hydrophilic character creates an optimized interface: hydrophobic NR blocks excess water penetration and hydrogen evolution, while polar PMMA facilitates Zn^2+^ transport and ion exchange.^[^ ^48^ ^]^ Such cooperative wetting regulation is key to enabling both stability and functionality at the Zn‐electrolyte interface.

### 2.5 Electrochemical Performance Across Wide Current Ranges

**Figure 5:**
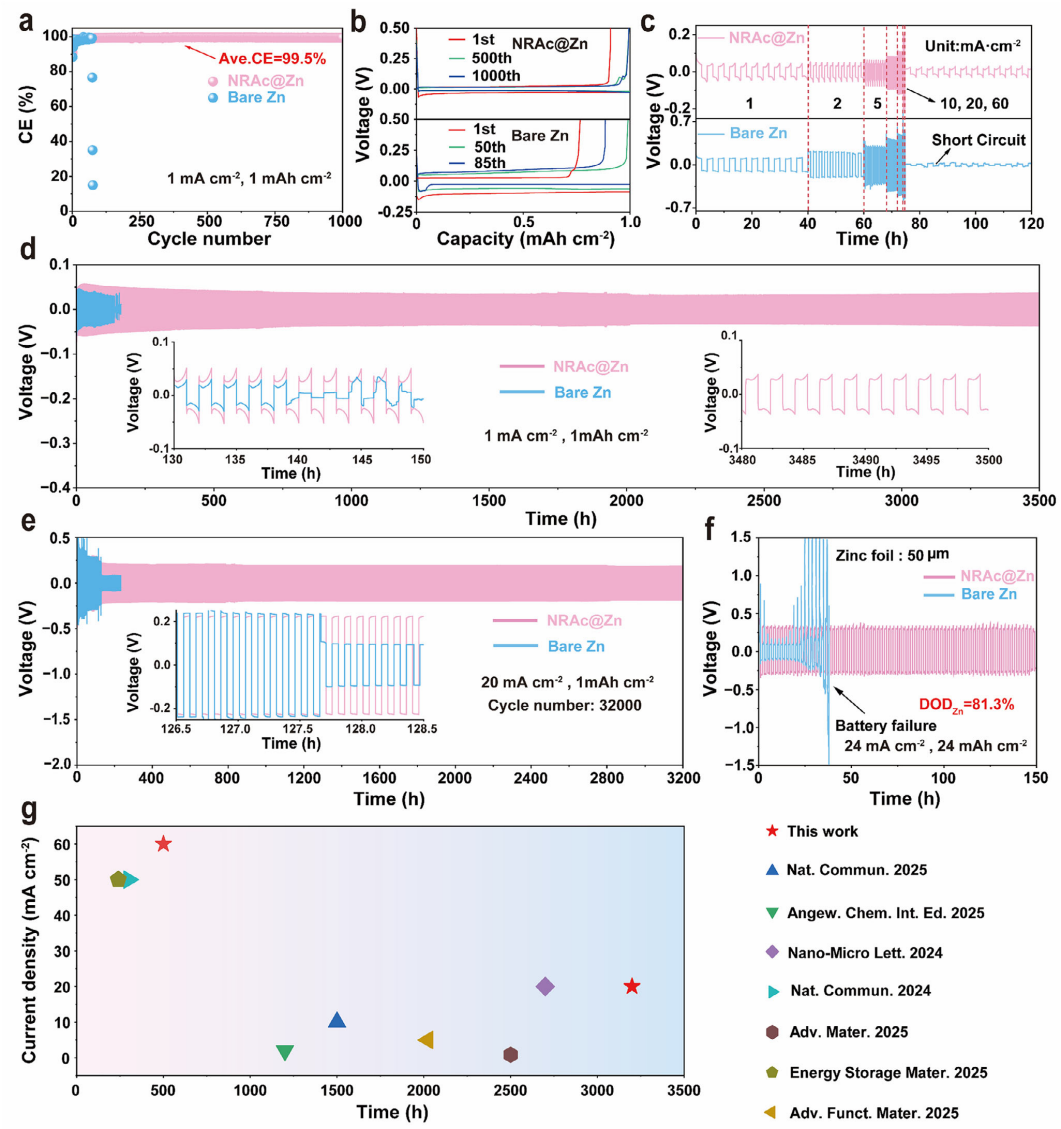
Electrochemical performance of Zn||Cu and Zn||Zn cells with and without NRAc coating. a) Coulombic efficiency of Zn||Cu cells at 1 mA cm^−2^, 1 mAh cm^−2^, showing rapid stabilization and long‐term retention. b) Representative plating/stripping profiles corresponding to panel a. c) Rate performance of symmetric Zn||Zn cells cycled at 1–60 mA cm^−2^. d) Long‐term cycling at 1 mA cm^−2^ and 1 mAh cm^−2^. e) Ultrastable cycling at 20 mA cm^−2^ and 1 mAh cm^−2^, exceeding 3200 h (≈32,000 cycles). f) Cycling with limited Zn supply (50 µm thickness, DOD_Zn_ = 81.3%) at 24 mA cm^−2^ and 24 mAh cm^−2^. g) Benchmark comparison of cycling stability with previously reported Zn||Zn cells.

The multifunctional design of NRAc translates directly into enhanced electrochemical reversibility. In Zn||Cu half‐cells cycled at 1 mA cm^−2^ and 1 mAh cm^−2^, the NRAc@Zn anode achieved rapid stabilization of Coulombic efficiency (CE) at ≈99% within 30 cycles and retained values above 99.5% for over 1000 cycles (**Figure** **5**a). By contrast, bare Zn degraded within 85 cycles due to unstable stripping and dendrite‐induced side reactions. The rapid CE stabilization of NRAc@Zn (≈99% within 30 cycles, >99.5% for >1000 cycles) aligns with recent advances showing that interfacial and structural control‐via electrolyte engineering‐can push CE to >99.5–99.9% and extend stable cycling into the thousands of hours.^[^ ^49^ ^]^ The corresponding voltage profiles (Figure 5b) illustrate the sharp difference in reversibility. Post‐mortem SEM and CV measurements (Figures S22 and S23, Supporting Information) further confirm that NRAc promotes smooth Zn deposition, whereas bare Zn surfaces display dendritic and heterogeneous morphologies. Potentiostatic polarization combined with EIS results (Figure S24, Supporting Information) prove that NRAc@Zn exhibits a zinc‐ion transference number (*t*_Zn_^2+^) of 0.63, significantly higher than that of bare Zn (*t*_Zn_^2+^ = 0.39; ≈62% increase). This confirms that the NRAc coating furnishes zincophilic transport channels that preferentially facilitate Zn^2+^ migration, thereby enhancing ionic transport efficiency. Similar improvements have been reported with polymeric interfacial layers such as polyacrylamide or poly(vinyl alcohol), though these typically stabilize CE for only a few hundred cycles before interfacial degradation occurs.^[^ ^50^, ^51^ ^]^

Symmetric Zn||Zn cells highlighted the coating's ability to sustain stable cycling across a remarkably wide current density window from 1 to 60 mA cm^−2^ (Figure 5c). This wide‐current stability is a critical yet elusive goal in zinc battery research, as different failure modes dominate at varying current densities.^[^ ^52^ ^]^ At moderate conditions (1 mA cm^−2^, 1 mAh cm^−2^), NRAc@Zn delivered over 3500 h of stable cycling (Figures 5d; S25, Supporting Information), a value that places it among the highest‐performing polymer‐based coatings reported to date.^[^ ^34^ ^]^ More notably, at the high current density of 20 mA cm^−2^, the NRAc layer enabled >3200 h of operation, corresponding to ≈32,000 plating/stripping cycles without failure (Figure 5e). This represents a substantial improvement over recent polymer‐based coatings, which typically report lifetimes on the order of tens to hundreds of hours at similar rates.^[^ ^52^ ^]^ Even under demanding conditions of limited Zn supply (50 µm foil, DOD_Zn_ = 81.3%) at 24 mA cm^−2^ and 24 mAh cm^−2^, a regime where high‐capacity operation is a known challenge,^[^ ^53^ ^]^ NRAc@Zn maintained stability for over 150 h, approximately three times longer than bare Zn (Figure 5f). At the extreme current density of 60 mA cm^−2^, NRAc@Zn further sustained cycling for more than 500 h (Figure S26, Supporting Information), highlighting the unprecedented robustness of the coating under a high‐rate, high‐capacity regime where most existing systems fail rapidly within tens of hours.^[^ ^52^ ^]^ A quantitative comparison with representative state‐of‐the‐art zinc anode modification strategies (Figure 5g and Table S1, Supporting Information) reveals that the NRAc coating achieves competitive or superior cycling stability across a wide current‐density range. Notably, at a high current density of 20 mA cm^−2^, its cycle life exceeds most reported counterparts under similar testing conditions. In addition, elevated‐temperature tests (60 °C) further confirm that NRAc affords markedly enhanced thermal stability compared with bare Zn (Figure S27 of the Supporting Information). We note that other advanced strategies‐such as metal‐organic framework (MOF) coatings or alloy interlayers‐have also demonstrated improved Zn cycling, but these approaches often involve complex synthesis or limited scalability.^[^ ^54^, ^55^ ^]^ By contrast, the NRAc coating benefits from its simple tape‐inspired design, scalable fabrication, and the synergy of elastic NR backbones with PMMA coordination sites (Figure 1a). Together, these results suggest that NRAc is an effective and practical means to achieve dendrite‐mitigated Zn cycling across an unprecedented operational window (1‐60 mA cm^−2^), although further testing under extended full‐cell conditions will be important to confirm long‐term scalability.

### 2.6 Device‐Level Demonstrations

**Figure 6:**
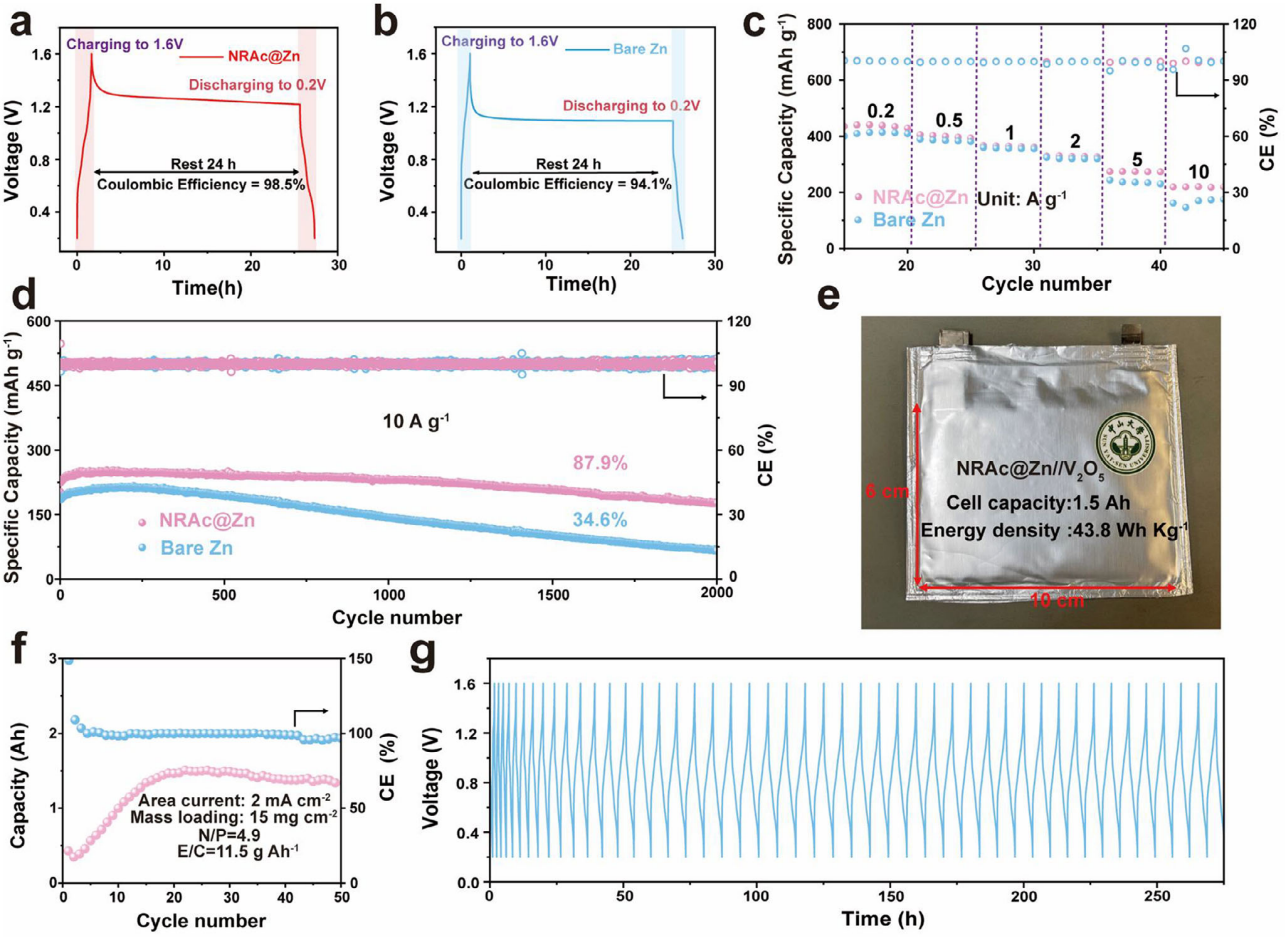
Zn||V_2_O_5_ full‐cell and pouch‐cell performance. a) Self‐discharge behavior of bare Zn|| V_2_O_5_ and b) NRAc@Zn||V_2_O_5_ coin cells after 24 h rest at 1.6 V. c) Rate capability at 0.2–10 A g^−1^. d) Long‐term cycling stability at 10 A g^−1^. e) Photograph of a 1.5 Ah NRAc@Zn||V_2_O_5_ pouch cell (6 × 10 cm^2^). f) Cycling stability and g) charge–discharge curves of the pouch cell at 2 mA cm^−2^.

To assess the practical applicability of NRAc modification, we evaluated V_2_O_5_||Zn full cells under both coin‐cell and pouch‐cell configurations. We first examined self‐discharge behavior to probe the extent of parasitic reactions at the Zn anode. Both bare Zn||V_2_O_5_ and NRAc@Zn||V_2_O_5_ coin cells were charged to 1.6 V, rested for 24 h, and then discharged to 0.2 V to quantify the remaining capacity. The bare Zn cell showed a notable capacity loss, corresponding to a coulombic efficiency of 94.1%, whereas the NRAc@Zn cell retained 98.5% efficiency after rest (**Figure** **6**a, b). This contrast highlights the ability of the NRAc coating to suppress parasitic processes such as Zn corrosion, thereby reducing self‐discharge‐consistent with recent reports that interface engineering and electrolyte/coating design markedly reduce anode corrosion and hydrogen evolution in practical Zn full cells.^[^ ^56^, ^57^ ^]^

The NRAc coating also improved rate capability. Both bare and coated anodes operated over a wide current density range of 0.2–10 A g^−1^, but the NRAc@Zn cells delivered markedly higher capacities at fast rates. The battery activated 15 times maintains ≈220 mAh g^−1^ at 10 A g^−1^, while bare zinc has a current of <200 mAh g^−1^ and shows unstable fluctuations during the cycling process (Figures 6c; S28, Supporting Information). Long‐term cycling stability at 10 A g^−1^ further underscored the benefit of NRAc: the modified anode retained 87.9% of its capacity after 2000 cycles, whereas the bare Zn anode suffered severe fading, retaining only 34.6% capacity under the same conditions (Figure 6d). In addition, EIS fitting analysis of the full cell further demonstrates that NRAc@Zn possesses a higher Zn^2+^ diffusion rate (Figure S29, Supporting Information), providing quantitative support for its superior high‐rate charge–discharge performance. These observations align with prior studies showing that polymeric or proton‐selective interlayers can enable fast Zn^2+^ transport while suppressing parasitic reactions, enabling high‐rate operation and extended cycling in Zn|| V_2_O_5_ full cells.^[^ ^57^, ^58^ ^]^

Encouraged by these promising results, we scaled up the system to pouch cells. A 1.5 Ah soft‐pack cell (6×10 cm^2^ electrodes, mass loading 15 mg cm^−2^, N/P ratio 4.9) was assembled using NRAc@Zn and V_2_O_5_ (Figures 6e; S30, Supporting Information). The pouch cell exhibited nearly complete capacity retention over 50 cycles at a current density of 2 mA cm^−2^ (Figures 6f, g). Further testing at 5 mA cm^−2^ confirmed the cell's stable performance (Figures S31 and S32, Supporting Information). Notably, even a single‐layer pouch cell was able to power a liquid‐crystal display (LCD) timer (Figure S33, Supporting Information), demonstrating the practical applicability of NRAc modification in real‐world energy storage applications. This pouch cell configuration achieved an E/C ratio of 11.5 g Ah^−1^ and a specific energy density of 43.8 Wh kg^−1^. This energy density is significantly higher compared to traditional lead‐acid batteries, which typically have an energy density ranging from 10–30 Wh Kg^−1^, highlighting the superior performance of this system.^[^ ^59^ ^]^ These scale‐up demonstrations align with recent studies that have successfully translated coin‐cell interface strategies into pouch‐cell prototypes.^[^ ^60^ ^]^

When placed in the broader context of recent reports, these results underscore the competitiveness of NRAc modification. Several polymer‐ and inorganic‐modified Zn anodes have demonstrated stable cycling in Zn||V_2_O_5_ full cells, but typically at moderate current densities (≤5 A g^−1^) or limited lifetimes (<1000 cycles).^[^ ^56^, ^57^, ^58^ ^]^ Reports of high‐current (≥10 A g^−1^) stability over thousands of cycles and reproducible Ah‐level pouch‐cell demonstrations remain scarce; the ability of NRAc to combine high‐rate operation (up to 10 A g^−1^), long‐term stability (>2000 cycles), and practical scalability (1.5 Ah pouch cells) therefore reflects the advantages of its tape‐inspired design which integrates elasticity, adhesion, and nanoconfined ion pathways.

### 2.7 Mechanistic Model

**Figure 7:**
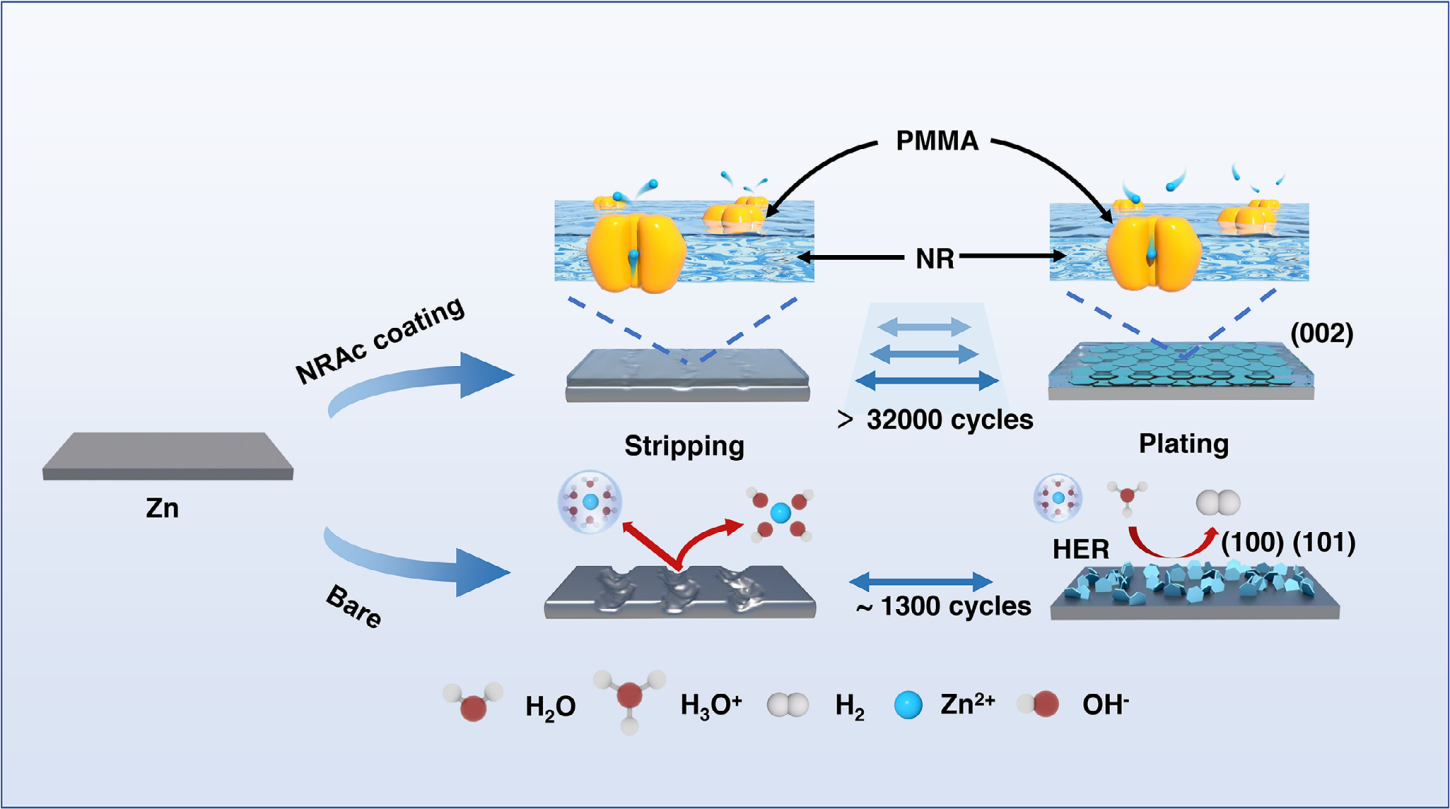
Schematic model of NRAc function. Nanoconfined Zn^2+^ transport channels and selective adsorption of PMMA segments bias Zn deposition toward the stable (002) plane, enabling dendrite‐mitigated, long‐term cycling.

The mechanistic insights gained from this study are summarized in **Figure** **7**. The NRAc coating unites tape‐inspired mechanical adhesion with nanoconfined Zn^2+^ transport pathways to achieve durable Zn anodes. The NR backbone contributes elasticity and hydrophobicity, ensuring conformal coverage on the rough Zn surface and serving as a physical barrier against electrolyte penetration. Simultaneously, PMMA side chains introduce polar carbonyl groups that selectively coordinate Zn^2+^, lower desolvation barriers, and guide deposition preferentially along the stable (002) crystallographic facet.

Similar mechanisms have been demonstrated in recent works: for example, an additive‐induced formation of Zn (002) texture suppresses dendrite growth and side reactions under high rates, highlighting the importance of facet control for durable Zn anodes.^[^ ^61^ ^]^ Polymer electrolyte systems bearing coordinating groups (e.g. ‐COO^−^) have been shown to reduce desolvation energy and improve Zn^2+^ ion transport, producing more uniform deposition and fewer parasitic reactions.^[^ ^56^ ^]^ Mechanical interlayers or coatings with strong adhesion and elasticity have also been reported to buffer volume changes and maintain conformal interphase coverage, thereby avoiding defects that enable dendritic protrusions or hydrogen evolution.^[^ ^62^ ^]^

As a result, the NRAc@Zn anode delivers remarkable stability across a wide current range (1–60 mA cm^−2^), including record lifetimes exceeding 32,000 cycles in symmetric cells and high‐rate operation up to 10 A g^−1^ in Zn||V_2_O_5_ full cells. The scalability of the approach was further demonstrated in 1.5 Ah pouch cells, where stable cycling performance confirmed the practical applicability of the coating. While these results highlight the significant advances enabled by NRAc, it is important to recognize that long‐term durability of Zn‐based pouch cells will also depend on the optimization of cathode architectures and electrolyte formulations. Our findings therefore establish NRAc as a robust and versatile anode‐protective layer, providing a foundation for further refinement toward commercial deployment of high‐performance aqueous Zn batteries.

## 3 Conclusion

We present a tape‐inspired PMMA‐grafted natural rubber coating that unites adhesion, elasticity and nanoconfined ion transport to stabilize zinc anodes. The NR matrix delivers conformal, mechanically adaptive protection while PMMA nanodomains coordinate Zn^2+^, lower desolvation energy and bias deposition toward the (002) facet. This combined mechanical‐chemical regulation suppresses dendrite formation and hydrogen evolution and yields exceptional electrochemical stability across a wide current range. NRAc@Zn symmetric cells exceed 32,000 stable cycles, Zn||V_2_O_5_ full cells retain long‐term performance at 10 A g^−1^, and a 1.5 Ah pouch prototype demonstrates scalability. More broadly, tape‐inspired microphase polymer coatings offer a transferable design principle for multifunctional protective layers on metal anodes in next‐generation energy storage.

## Conflict of Interest

The authors declare no conflict of interest.

## Author Contributions

P.Z. planned and designed the project that was discussed with X.Y., X.H., Z.Z., and D.S. S.Z., S.L., Y. Z., R.X., and X.Y. fabricated the materials and performed the electrochemical experiments. Z.L., X.L., S.L., and J.L. did the structure analysis. S.Z. and P.Z. analyzed the data. P.Z. and S.Z. wrote the manuscript. All authors discussed the results and commented on the manuscript.

## Supporting information



Supporting Information

Supporting Information

Supporting Information

## Data Availability

The data that support the findings of this study are available from the corresponding author upon reasonable request.
