# Supplementary material for: Adhesive Tape‐Inspired Polymer Coatings Enable Record‐Stable Zinc Anodes for High‐Rate Aqueous Batteries
Source: Adv Sci (Weinh). 2025 Dec 12;13(10):e20648. doi: 10.1002/advs.202520648 (PMC12915122; doi:10.1002/advs.202520648)
Supplement: Supplementary file 1 — Supporting Information [file ADVS-13-e20648-s001.docx]

Supporting Information

Adhesive Tape-Inspired Polymer Coatings Enable Record-Stable Zinc Anodes for High-Rate Aqueous Batteries

Shuo Zhao^a^, Shuyue Luo^a^, Yangming Zhang^a^, Rongyan Xie^a^, Zhen Liu^b^, Zhengyin Yao^a^, Xianru He^c^, Jiangbo Li^d^, Xiang Yao^e^, Zhou Zhou^f^, Dongbai Sun^a^, Peng Zhang^*,a^

a. School of Materials Science and Engineering, Key Laboratory for Polymeric Composite and Functional Materials of Ministry of Education, Southern Marine Science and Engineering Guangdong Laboratory (Zhuhai), Institute of Green Chemistry and Molecular Engineering, Sun Yat-sen University, Guangzhou 510275, China

b. Medical Devices Research & Testing Center, South China University of Technology, Guangzhou 510006, China

c. School of New Energy and Materials, Southwest Petroleum University, Chengdu 610500, China

d. Engineering Center for Superlubricity, Jihua Laboratory, Foshan 528200, China

e. JiangSu CheeShine Performance Materials Co., Ltd., Huaian 223200, China

f. Center for Neutron Science and Technology, Guangdong Provincial Key Laboratory of Magnetoelectric Physics and Devices, School of Physics, Sun Yat-sen University, Guangzhou 510275, China

^*^Corresponding author: [zhangpeng3@mail.sysu.edu.cn](mailto:zhangpeng3@mail.sysu.edu.cn) (P.Z.)

**Experimental Section**

**Materials**. The grafted polymer NRAc (natural rubber grafted with PMMA, PMMA ≈ 30 wt%) was supplied by Jiangsu Qixiang High-tech Materials Co., Ltd. Zinc foil (50 μm and 100 μm) and stainless-steel foil (10 μm) were purchased from Nuo Tai Metal Materials Company. Conductive acetylene black (Super P), polyvinylidene fluoride (PVDF, HSV900, >99%) and copper foil (10 μm) were obtained from Canrd New Energy Technology. Sandpaper (Eagle Brand, 1000 grit) was used for Zn polishing. Zinc trifluoromethanesulfonate (Zn(OTf)_2_, >98.0%) was purchased from Shanghai Deepbio Biotechnology Co., Ltd., tetrahydrofuran (THF, >99.9%) from Shanghai Macklin Biochemical Co., Ltd., and vanadium pentoxide (V_2_O_5_, >99%), zinc sulfate heptahydrate (ZnSO_4_·7H_2_O, >99.9%), and N-methyl-2-pyrrolidone (NMP, >99.0%) from Aladdin. Glass microfiber separators (GF/D, 2.7 μm) were obtained from Whatman, and CR2025-type coin cell components from Guangdong Canrd New Energy Technology Co., Ltd.

**NRAc@Zn anode preparation**. NRAc powder (0.10 g) was dissolved in 5 mL THF and magnetically stirred for 24 h. Zinc foil was polished with 1000-grit sandpaper to expose a fresh surface, after which the solution was spin-coated (KW-4A, Institute of Microelectronics, CAS) at 600 rpm for 5 s followed by 4000 rpm for 35 s. The coated foils were dried at 80 °C for 60 min to remove residual solvent.

**V_2_O_5_ cathode preparation**. V_2_O_5_, Super P and PVDF were mixed in a 7:2:1 weight ratio with NMP to form a slurry. The slurry was cast on stainless-steel foil with a 150 μm doctor blade and dried under vacuum at 60 °C overnight. For coin cells, electrodes with 1-2 mg cm^-2^ loading were punched into 12 mm disks. For laminated cells, cathodes were cut into 6 × 10 cm pieces with a loading of ~15 mg cm^-2^.

**Cell assembly**. CR2025 coin cells and laminated pouch-type cells were assembled under ambient conditions. Zn//Cu asymmetric and Zn//Zn symmetric cells used 2 M ZnSO_4_ electrolyte, while Zn//V_2_O_5_ full cells used 2 M Zn(OTf)_2_. Symmetric cells employed Zn foils (100 μm) unless stated otherwise. Laminated cells were assembled by stacking NRAc@Zn or bare Zn foils (6.5 × 10.5 cm, 50 μm thick), cathodes (6 × 10 cm), and separators (7 × 11 cm) with 10 mL electrolyte, followed by heat sealing.

**Electrochemical measurements**. CV, EIS, Tafel and potentiostatic polarization were performed on a CHI660E electrochemical workstation. Galvanostatic charge–discharge was conducted using a LANHE CT2001A system over 0.2-1.6 V. EIS spectra were recorded from 100 kHz to 0.01 Hz. Tafel analysis used a two-electrode configuration with Zn or NRAc@Zn as the working electrode and Cu foil as the counter electrode. Coulombic efficiency was determined with a cut-off stripping potential of 0.5 V. Zn^2^⁺ transference numbers were measured using the Bruce–Vincent method from potentiostatic polarization (-150 mV, 400 s) and pre/post EIS on Zn//Zn symmetric cells.

**In-situ pH monitoring**. Zn//Zn symmetric cells were cycled at 10 mA cm^-2^ with a 10 min plating/stripping duration while local pH near the electrode surface was monitored using a PH-100 probe (LICHEN).

**Mechanical testing**. Mechanical properties of NRAc films were measured on a CMT4504 universal testing machine. Tensile specimens (4×25 mm) and peel specimens (1 × 5 cm) were prepared, with peel tests conducted at 180° after rolling three times on a roller press (MSK-2150, Shenzhen KeJing Star Technology Co.). Crosshead speeds were 20 mm min^-1^ and 25 mm min^-1^ for tensile and peel tests, respectively, according to ASTM standards.

**Materials characterization**. Sample morphologies and elemental distributions were examined by SEM (Gemini 500, Zeiss). Crystallinity was characterized by XRD and WAXS (D8 Advance, Bruker), with 2D WAXS patterns acquired under vacuum at 300 s exposure. SAXS was performed at the BioSAXS BL19U2 beamline of the Shanghai Synchrotron Radiation Facility using a PILATUS 1M detector (λ = 1.033 Å, sample–detector distance 2672 ± 1 mm); samples were sealed with Scotch tape, kept under nitrogen, and processed with RAW software (v2.3.1) ^[1]^. Surface roughness was evaluated by CLSM (VHX-X1, Keyence), while TEM images were acquired on a JEM-1400Plus (JEOL, 200 kV). TOF-SIMS was conducted on an ION-TOF IV instrument with a pulsed 30 keV Ga source in negative mode. MALDI-TOF and FTIR spectra (Nicolet IS50, Thermo Fisher, ATR mode) were also collected. All measurements were carried out at 25 ± 1 °C unless otherwise stated.

**Theoretical calculation**. Finite element simulations were performed using COMSOL Multiphysics 6.2 (“cubic current distribution” module) to analyze electric field and Zn^2^⁺ concentration profiles. Ion transport was described by the Nernst-Planck equation:

$$\frac{\partial c_{i}}{\partial t}=\nabla\bullet\left( D_{i}\nabla c_{i}+z_{i}\frac{D_{i}}{RT}Fc_{i}\nabla_{\phi} \right)$$

where, $c_{i}$ is the concentration of electrolyte ion i; $t$ is the time variable (appearing in the continuity equation); $D_{i}$ is the diffusion coefficient of ion i; $Z_{i}$ is the charge number of ion i; $F$ is the Faraday constant; $\phi$ is the electrostatic potential; $R$ is the universal gas constant and $T$ temperature. The model considered two parallel electrodes (10 μm long, 7 μm apart) immersed in 2 M Zn^2⁺^ electrolyte (diffusion coefficient 8×10^−16^ m^2^ s^-1^). The positive electrode was set to zero potential, and the negative electrode was biased at the cell polarization potential. Exchange current density was 1 mA cm^-2^ at 298 K. Density functional theory (DFT) calculations were conducted within the VASP framework using the PBE-GGA functional. Zn (002), (101), and (001) facets were modeled to evaluate adsorption energetics. A two-step relaxation scheme was applied: (i) coarse optimization with a 450 eV cutoff and 4 × 4 × 1 k-point mesh (SCF convergence 10^−5^ eV, ionic relaxation -0.2 eV Å^-1^); and (ii) refined optimization with a 560 eV cutoff, convergence 5×10^-7^ eV for SCF, and -0.02 eV Å^-1^ for ionic forces. Adsorption energies were extracted from fully relaxed slab-adsorbate systems.


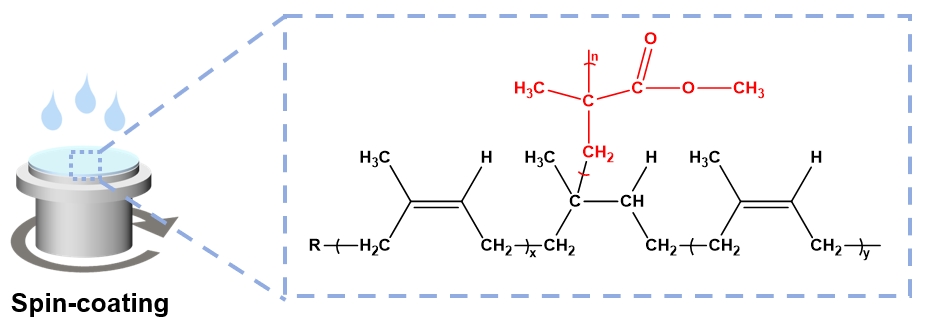


**Figure S1.** Schematic illustration of the preparation process of the NRAc@Zn electrode, showing uniform coating of NRAc on polished Zn foil prior to electrochemical assembly.


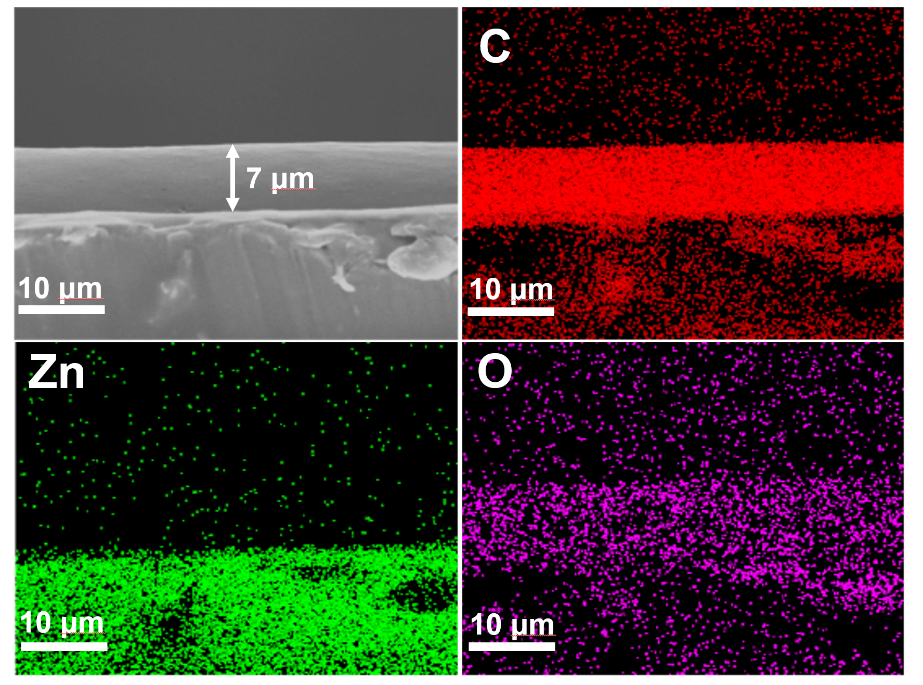


**Figure S2.** Cross-sectional FESEM image and corresponding elemental mapping of NRAc@Zn, confirming intimate adhesion of the NRAc coating and homogeneous elemental distribution across the Zn interface.


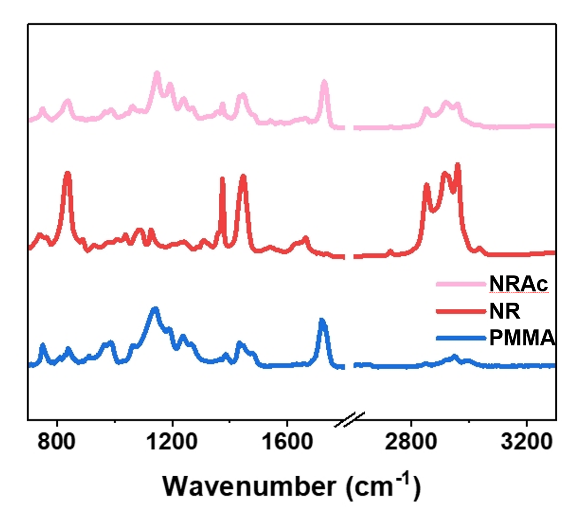


**Figure S3.** FTIR spectra of NRAc, NR, and PMMA. The characteristic carbonyl stretching peak of PMMA and hydrocarbon vibrations of NR verify the coexistence of polar and elastic components within NRAc.


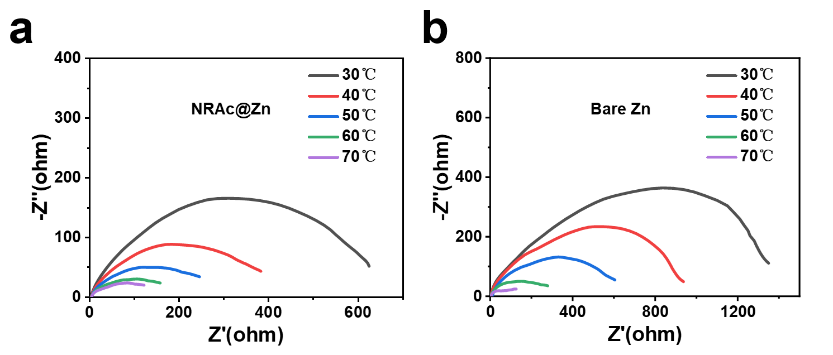


**Figure S4.** EIS spectra of a) NRAc@Zn and b) bare Zn electrodes at various temperatures. Arrhenius analysis yields activation energies of 33.53 kJ mol^-1^ (NRAc@Zn) and 52.82 kJ mol^-1^ (bare Zn), consistent with Figure 2c.


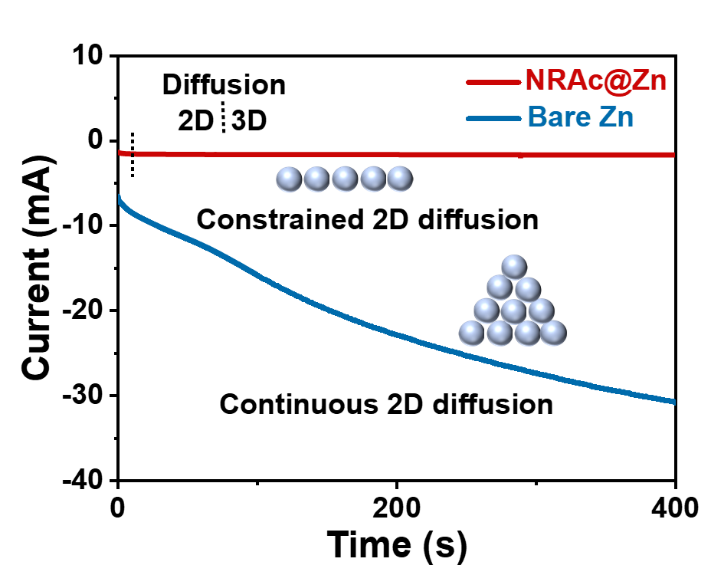


**Figure S5.** Chronoamperometry of Zn||Zn symmetric cells at -150 mV overpotential. When the overpotential is set to150mV, the current density of bare Zn symmetrical cell increases continuously within 400s, showing a typical 2D nucleation diffusion process. Typically, this diffusion mode may lead to aggregation and dendrite growth, similar to the tip effect.


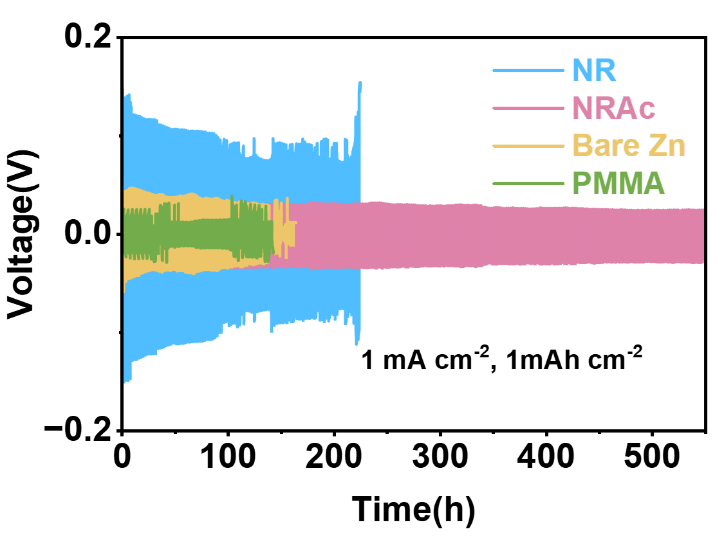


**Figure S6.** Cycling performance of symmetric cells assembled with NRAc, NR-only, or PMMA-only films at 1 mA cm^-2^ and 1 mAh cm^-2^. NRAc exhibits the best stability, demonstrating the synergistic effect of NR elasticity and PMMA coordination.


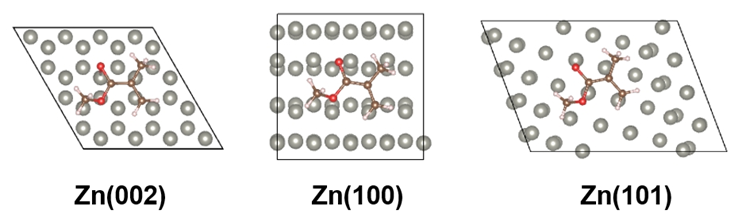


**Figure S7.** DFT adsorption models of MMA molecules on Zn (002), Zn (100), and Zn (101) surfaces. Adsorption energies are weakest on (002) (-0.11 eV), consistent with preferential (002) growth shown in Figures 3a and b.


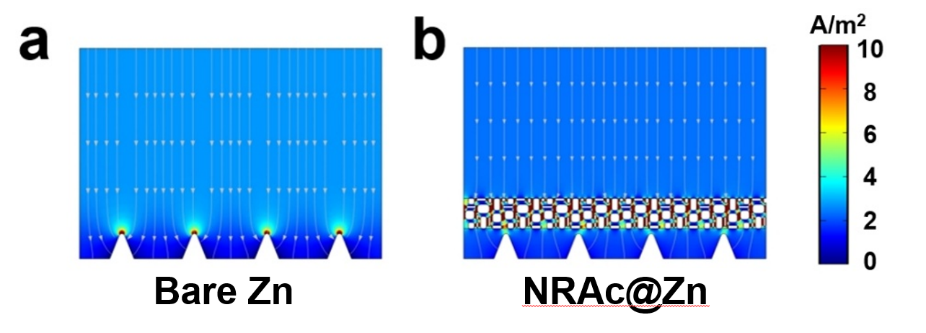


**Figure S8.** COMSOL simulations of Zn plating. a) Bare Zn exhibits localized high-field regions. b) NRAc@Zn displays uniform electric field and Zn^2+^ concentration distribution, supporting in situ optical results in Figure 3e.


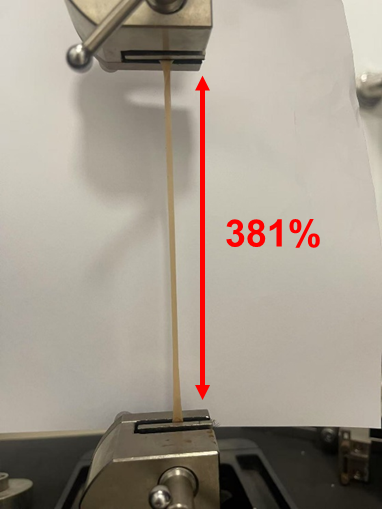


**Figure S9.** Photograph of NRAc film stretched to 381% elongation on a universal tensile tester, confirming high elasticity. Corresponds to stress-strain behavior in Figure 4a.


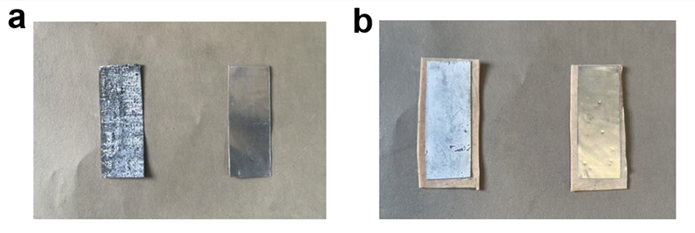


**Figure S10.** Optical images of Zn surfaces: a) bare Zn before and after deposition showing cracks; b) Zn surface conformally coated with NRAc, showing smooth and intact morphology. Supports peel-strength results in Figures 4c and d.


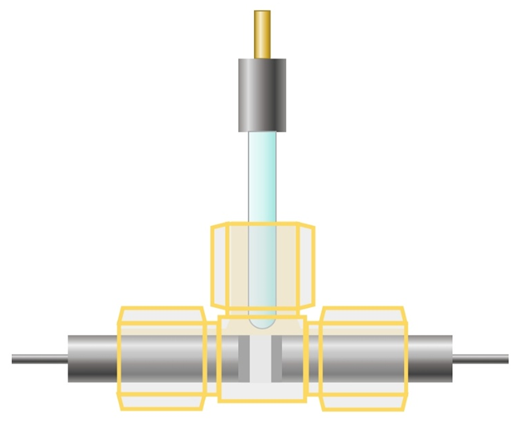


**Figure S11.** Schematic diagram of the Swagelok-type cell used for Tafel measurements.


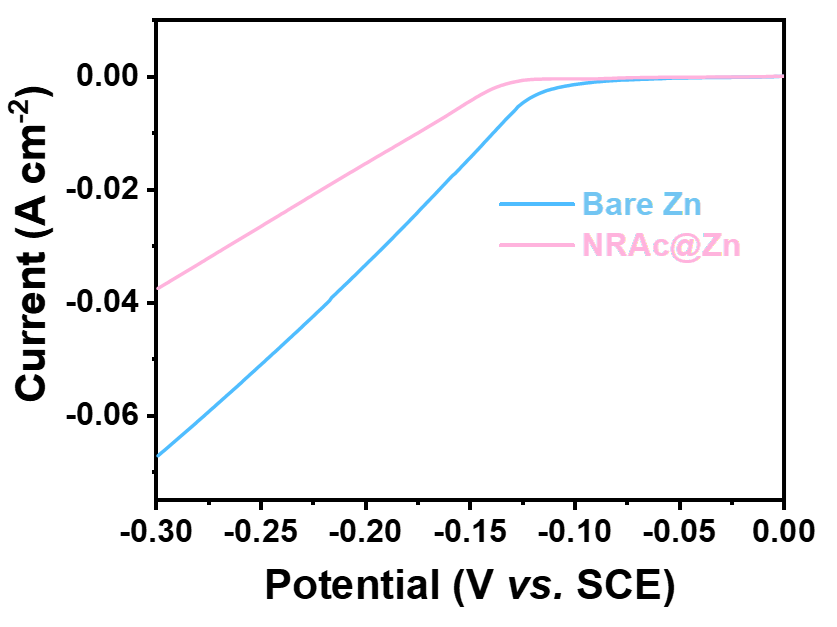


**Figure S12.** Linear sweep voltammetry (LSV) curves of bare Zn and NRAc@Zn electrodes at 1 mV s^-1^. NRAc@Zn shows higher hydrogen-evolution overpotential, consistent with reduced corrosion (Figure 4f).


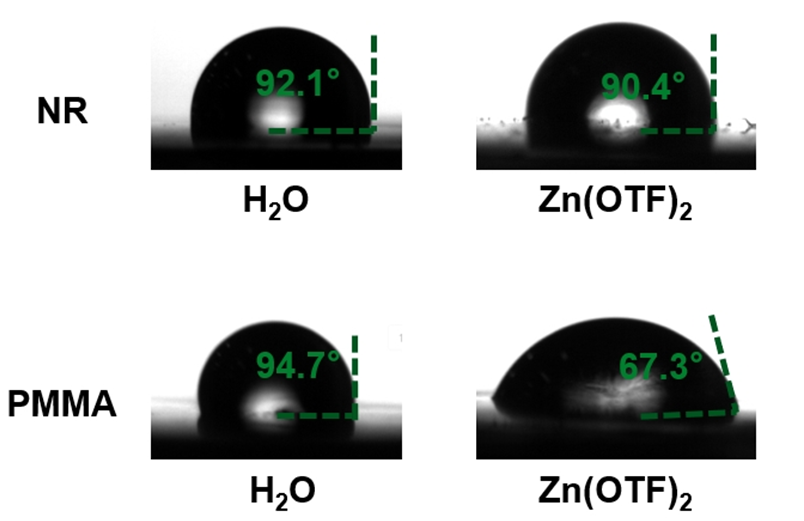


**Figure S13.** Contact angles of water and 2 M Zn(OTf)_2_ electrolyte on NR and PMMA films. NR is hydrophobic toward both, while PMMA shows lower angles with electrolyte, supporting the dual hydrophilic–hydrophobic balance in NRAc (Figure 4i).


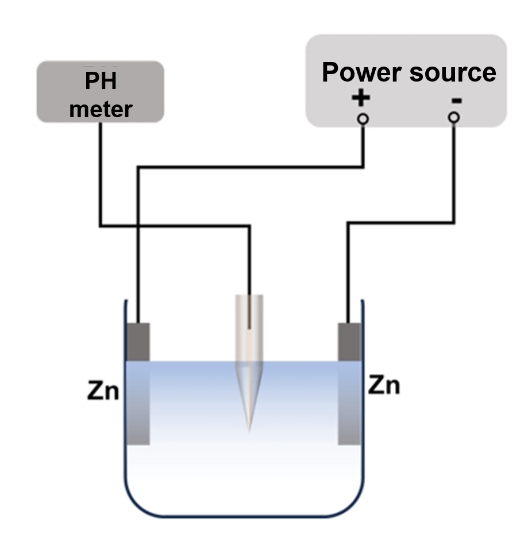


**Figure S14.** Schematic diagram of the *in situ* pH-monitoring device for symmetric cells.


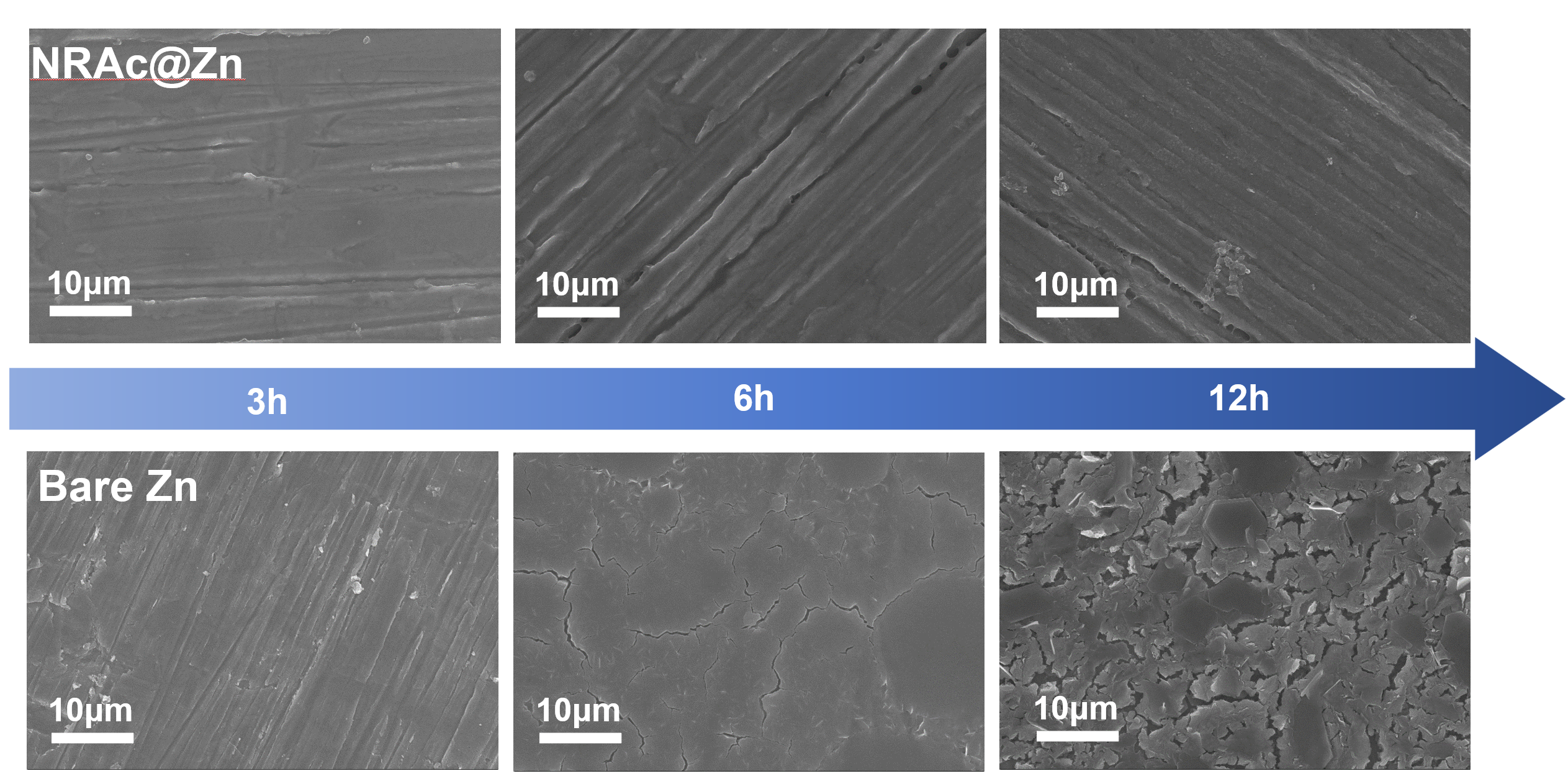


**Figure S15.** FESEM images of bare Zn and NRAc@Zn after immersion in 2 M Zn(OTf)₂ solution for different durations. Bare Zn develops cracks after 6-12 h, while NRAc@Zn remains smooth, supporting pH and corrosion-resistance data (Figure 4f).


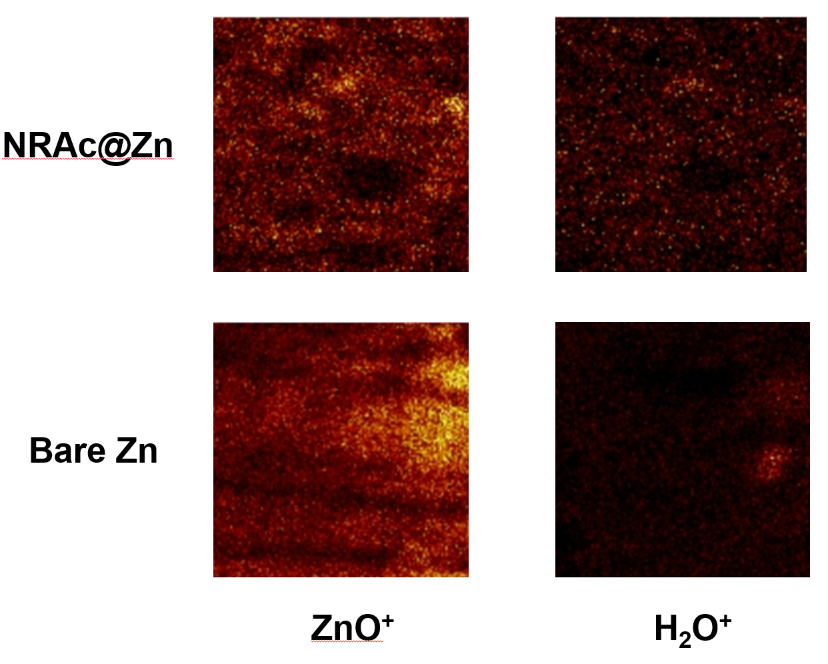


**Figure S16.** TOF-SIMS surface maps of cycled Zn anodes, showing spatial distributions of ZnO⁺ and H₂O. Bare Zn shows high-intensity byproduct signals, while NRAc@Zn suppresses their formation, consistent with Figure 4h.


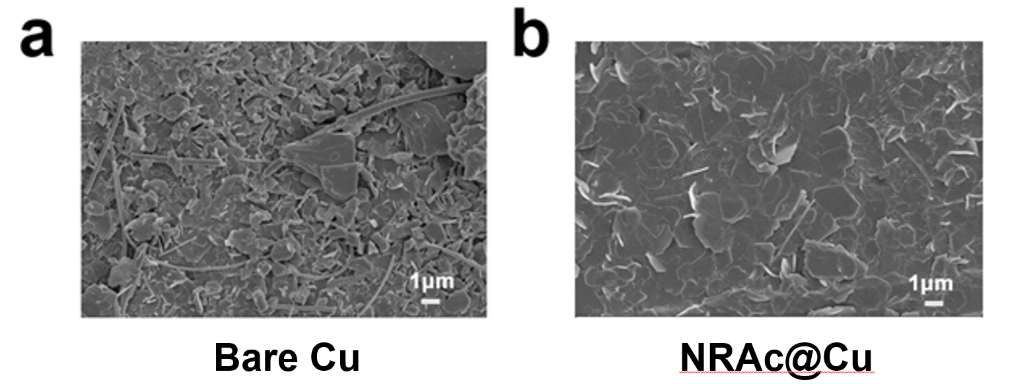


**Figure S17.** Surface SEM images of Cu electrodes cycled against bare Zn and NRAc@Cu anodes. Severe dendritic Zn deposition is observed with bare Zn, whereas NRAc@Cu yields smooth, uniform deposition. Supports Coulombic efficiency results in Figure 5a and b.


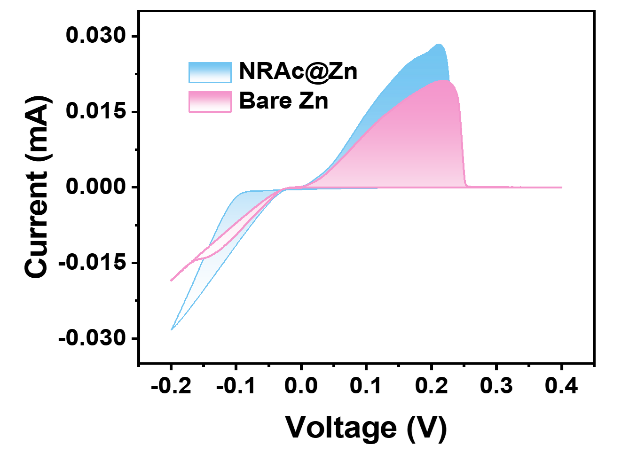


**Figure S18.** Cyclic voltammetry (CV) curves of Zn||Cu half-cells with bare Zn and NRAc@Zn anodes at 1 mV s^-1^. NRAc@Zn shows lower overpotentials and higher reversibility, consistent with improved CE in Figure 5a.


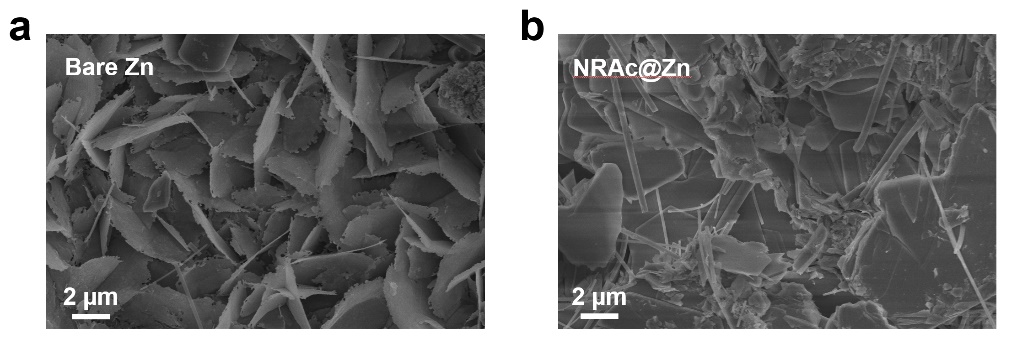


**Figure S19.** FESEM images of (a) bare Zn and (b) NRAc@Zn electrodes after long-term cycling. NRAc suppresses dendritic growth and surface cracks, consistent with stability results in Figure 5d and e.


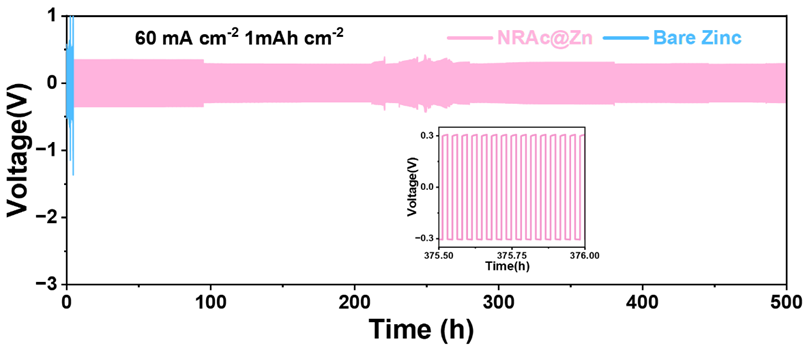


**Figure S20.** Galvanostatic cycling of Zn||Zn symmetric cells at 60 mA cm^-2^ and 1 mAh cm^-2^. NRAc@Zn maintains stability for >500 h, whereas bare Zn fails rapidly.


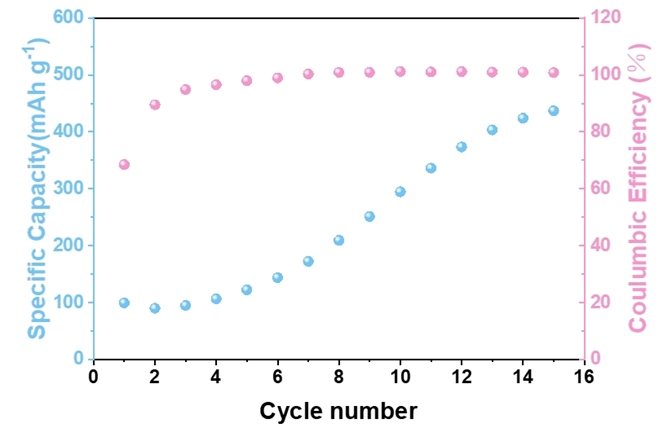


**Figure S21.** Initial 15 activation cycles of V₂O₅ cathodes at 0.2 A g^-1^. Used prior to long-term cycling tests in Figure 7d.


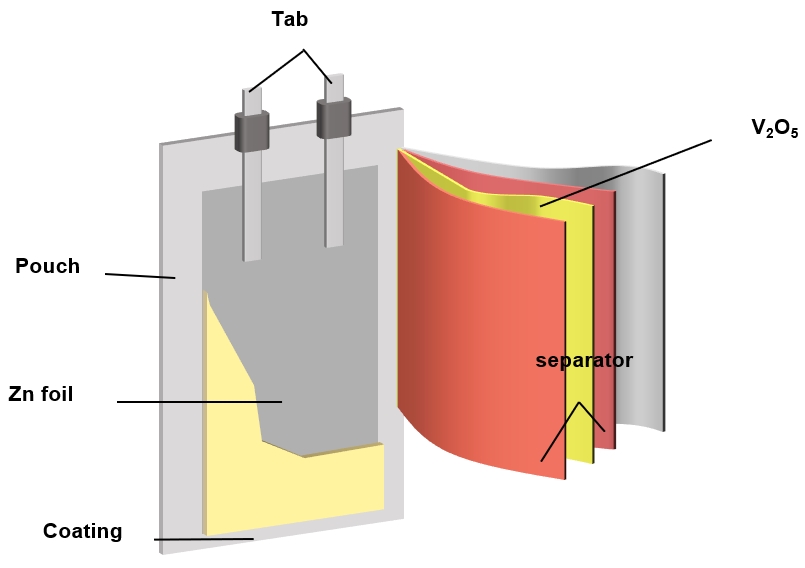


**Figure S22.** Stepwise assembly process of the 1.5 Ah NRAc@Zn||V₂O₅ pouch cell (6 × 10 cm² electrodes).

**Figure S23**. Cycling performance of pouch cells at 5 mA cm^-2^, showing stable charge-discharge capacity retention.

**Figure S24**. Charge-discharge voltage profiles of pouch cells at 5 mA cm^-2^. Profiles remain stable after extended cycling.


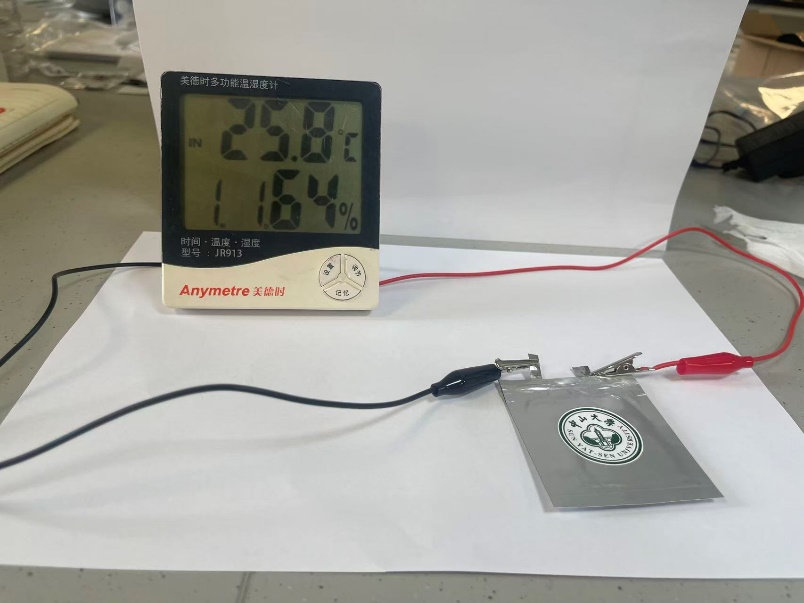


**Figure S25**. Demonstration of a single-layer NRAc@Zn||V₂O₅ pouch cell powering an LCD timer, highlighting potential for practical device applications.

**Reference**

[1] N. Li, X. Li, Y. Wang, G. Liu, P. Zhou, H. Wu, C. Hong, F. Bian, R. Zhang, *J Appl Crystallogr* **2016**, *49*, 1428–1432.

[2] C. Huang, X. Zhao, Y. Hao, Y. Yang, Y. Qian, G. Chang, Y. Zhang, Q. Tang, A. Hu, X. Chen, *Energy Environ. Sci.* **2023**, *16*, 1721–1731.
